# ClpP deficiency attenuates contrast-induced HK-2 cell injury through changes associated with mitochondrial dynamics and apoptosis

**DOI:** 10.1371/journal.pone.0352422

**Published:** 2026-07-02

**Authors:** Jing Wang, Lei Wang, Liang Xie, Chengchun Tang

**Affiliations:** 1 School of Medicine, Southeast University, Nanjing, Jiangsu, China; 2 Department of General Medicine, Women’s Hospital of Nanjing Medical University. Nanjing Women and Children’s Healthcare Hospital, Nanjing, Jiangsu, China; 3 Jinling Hospital Department of Cardiology, Nanjing University, School of Medicine, Nanjing, Jiangsu, China; 4 Department of Cardiology, Zhongda Hospital of Southeast University Medical School, Nanjing, Jiangsu, China; INSERM, FRANCE

## Abstract

**Background:**

Contrast-associated acute kidney injury (CA-AKI) is a renal impairment that occurs after several days of intravascular administration of iodine-containing contrast media. ClpP is a key protease that plays an important role in cellular mitochondrial function. This study investigated the role of ClpP in mitochondrial dynamics and early injury in an in‑vitro CA‑AKI model.

**Methods:**

mRNA sequencing was performed on HK-2 cells with or without iohexol exposure. Cell viability, mitochondrial dynamics-related protein expression, mitochondrial membrane potential (MMP), and cell apoptosis were assessed by cell counting kit-8, immunoblotting, JC‑1 staining and flow cytometry, respectively.

**Results:**

Iohexol treatment at 80 mg I/mL reduced HK-2 cell viability to 63.44%, induced mitochondrial fission, inhibited mitochondrial fusion and promoted apoptosis. mRNA sequencing revealed significant upregulation of *Opa1* and *ClpP* gene expression, as well as alterations in proteasome‑related signaling in iohexol-induced HK-2 cell. Western blot analysis further confirmed elevated ClpP protein expression after iohexol exposure. Importantly, ClpP knockdown partially restored MMP, increased Opa1 expression, improved mitochondrial morphology, and alleviated iohexol‑induced apoptosis.

**Conclusion:**

ClpP deficiency may exert cytoprotective effects against iohexol-induced HK-2 cell injury, at least partly through changes associated with mitochondrial dynamics, partial preservation of MMP, and attenuation of apoptosis. These findings suggest that ClpP may represent a potential molecular target for further investigation in CA-AKI.

## 1. Introduction

Acute kidney injury (AKI) is a sudden decline in kidney function that results in retention of urea and other nitrogenous wastes as well as dysregulation of extracellular fluid volume and electrolytes. In developed countries, AKI occurs mainly in hospitalized elderly patients and is associated with sepsis, drugs, and invasive procedures [1]. In the present study, we focused on contrast-associated acute kidney injury. With the increasing incidence of cardiovascular disease and the development of coronary angiography, the use of iodinated contrast agents is becoming more widespread, and some patients may develop contrast-associated acute kidney injury (CA-AKI) within days after intravascular administration of iodinated contrast media [2]. CA-AKI has been widely recognized as a major cause of hospital-acquired AKI [3]. However, there is no effective clinical treatment, and only certain preventive measures can be taken against CA-AKI [4]. Therefore, there is an urgent need to explore novel therapeutic options to alleviate renal injury and improve patient outcomes.

The pathogenesis of CA-AKI is complex, and recently, several studies have suggested that direct nephrotoxicity, renal ischemia, oxidative stress, mitochondrial damage, and mitosis are involved in the pathogenesis of CA-AKI [5 - 7]. However, the exact mechanism of CA-AKI is not fully clear. Thus, it is crucial to study the molecular mechanisms and explore molecular targets of CA-AKI for early diagnosis and intervention. This may involve developing effective treatment strategies and improving patient prognosis.

Inflammation is crucial in the pathogenesis of CA-AKI [8]. Inflammatory stimuli could cause mitochondrial dysfunction, result in ATP consumption, increase ROS production, calcium dysregulation, mitochondrial pore formation, pro-apoptotic protein release, and apoptosis [9]. Mitochondria provide energy for cellular metabolism by constantly dividing and fusing under pathological conditions. This process is known as mitochondrial dynamics [10]. Mitochondrial dynamics refers to the fact that mitochondria in a cell are in a constant state of division and fusion, which is co-regulated by fission-related proteins (DRP1, FIS1) and fusion-related proteins (MFN1, MFN2, OPA1) [11]. A study found that Tolvaptan could improve mitochondrial function by inhibiting DRP1 expression and increasing MFN2 expression in CA-AKI rats [12]. Subsequent in vivo studies have also demonstrated that mitochondria in proximal renal tubule cells undergo fragmentation during ischemia/reperfusion and cisplatin-induced acute kidney injury in the mouse kidney [13]. Accordingly, mitochondrial dynamics may play a crucial regulatory role in CA-AKI, and warrant further investigation.

Caseinolytic peptidase P (ClpP) is an ATP-dependent protease, which is located in the mitochondrial matrix [14]. ClpP plays an essential role in cellular homeostasis and metabolic regulation by degrading misfolded or damaged proteins and maintaining mitochondrial proteostasis [15, 16]. ClpP is closely associated with a series of processes, including the life-span of cells, mitochondrial fusion and fission, DNA repair, and apoptosis [17]. Abnormalities in HsClpP lead to mitochondrial dysfunction, which may contribute to a variety of diseases, such as neurological disorders, tumors, metabolic syndromes, diabetes, gastrointestinal disorders, and so on [15, 18, 19]. The loss of ClpP enhanced mitochondrial division and inhibited mitochondrial fusion were observed in the high-glucose and high-fat induced mice islet β-cell line Min6 [20]. ClpP deficiency causes mitochondrial dysfunction and decreases cell proliferation in muscle cells [21]. However, few studies have been reported on the role and molecular mechanisms of ClpP in CA-AKI. Exploring the role and regulatory mechanisms of ClpP is important for revealing the pathogenesis of CA-AKI and finding novel therapeutic targets.

Therefore, based on the potential regulatory role of ClpP in mitochondrial homeostasis, the present study aimed to investigate whether ClpP is involved in iohexol-induced HK-2 cell injury and to explore whether this effect is associated with mitochondrial dynamics, mitochondrial ultrastructure, MMP changes, and apoptosis. These findings may provide further insight into the role of ClpP-related mitochondrial changes in CA-AKI.

## 2. Materials and methods

### 2.1. Cell culture and treatment

HK-2 cell was purchased from the American Type Culture Collection (ATCC). Cells were cultured in Dulbecco’s modified Eagle’s medium (DMEM/F12) containing 10% fetal bovine serum (FBS, # 1943609, Sigma, China) and 1% penicillin-streptomycin (# C0222, Beyotime, Jiangsu, China). When the cells were in a good state of growth, they were randomly divided into two groups: the CA-AKI group and the control group. The in-vitro CA-AKI model was established using an appropriate concentration of iohexol. The concentrations of iohexol were determined by CCK8 experiments.

To knock down the target gene *ClpP*, HK-2 cells were treated with lentiviral transduction enclosing ClpP shRNA (LV-sh-ClpP) (# 124353−1, Shanghai GeneChem Co., Ltd) according to the manufacturer’s instructions. shRNA against ClpP (sh-ClpP) and the negative control shRNA (sh-NC, Scramble) were transfected into HK-2 cells, respectively, and the knockdown efficiency was analyzed by RT-qPCR and Western blot.

### 2.2. Cell viability analysis

HK-2 cell viability was detected by the Cell Counting Kit-8 (CCK8) assay (# C0037, Beyotime, Jiangsu, China) according to the manual. Briefly, HK-2 cells were cultured in 96-well plates to make 1 × 10^4^ cells per well and incubated at 37°C in a CO2 incubator. When the cells reached 80% confluence, before cell injury, cells were serum-starved for 6 h and then treated with iohexol at doses of 5, 10, 20, 40, 80, 160, and 320 mg I/ml for 2 h (Iohexol, # 74147, Sigma, China). Subsequently, 10 μL CCK-8 reagent was added to each well and incubated for about 1–4 h before measuring the absorbance of the cells at 450 nm. HK-2 cells were treated with DMEM/F12 medium and cell-free medium were used as negative and blank controls, respectively. Cell viability was evaluated as a percentage of the absorbance of the treated group compared to the control group.

### 2.3. mRNA sequencing and data analysis

We performed high-throughput sequencing on HK-2 cells with or without iohexol treatment (n = 3 in each group). mRNA sequencing was conducted by Shanghai Biotechnology Corporation (Shanghai, China) using the Illumina NovaSeq6000 platform. Total RNA was obtained from HK-2 cells using Trizol reagent (# R411-01, Vazyme Biotech, China). RNA integrity was examined by an Agilent 2100 Bioanalyzer (Agilent Technologies). The concentration and purity of total RNA were measured using a Qubit 2.0 Fluorescence Quantimeter (Thermo Fisher Scientific) and a NanoDrop ND-2000 Spectrophotometer (Thermo Fisher Scientific). Purified total RNA was used to construct mRNA sequencing libraries. Library concentration was measured with a Qubit 2.0 Fluorescence Quantifier (Thermo Fisher Scientific) and library fragment distribution was measured with an Agilent 4200 TapeStation (Agilent Technologies). Sequencing was carried out in accordance with the effective concentration of the library and data output requirements.

We calculated the number of fragments for each gene after Hisat2 comparisons using Stringtie (version: 1.3.0) [22, 23], then normalized them using the TMM (Trimmed Mean of M values) method [24], and ultimately calculated the FPKM value for each gene using a script. Genes with an average FPKM> 1 were used for subsequent scatterplot analyses. Differential expression analysis was conducted by DESeq2, and genes with adjusted P values < 0.05 were regarded as differentially expressed.

Clusters of expression patterns were produced by unsupervised hierarchical cluster analysis and K-means clustering algorithms using the R. DAVID, and revealed by Gene Ontology (GO) and Kyoto Encyclopedia of Genes and Genomes (KEGG) analyses using Ingenuity Pathways Analysis (IPA). mRNA sequencing experiments were carried out in three biological replicates.

### 2.4. Transmission Electron Microscopy (TEM)

HK-2 cells treated with or without 80 mg I/mL of iohexol for 2 h were fixed in 2.5% glutaraldehyde solution (# 111-30-8, SPI Supplies, American). Subsequently, the samples were processed according to the standard protocol, including dehydration, embedding, and sectioning, and were ultimately observed using a Hitachi 7500 transmission electron microscope (Hitachi, Tokyo, Japan).

### 2.5. Determination of mitochondrial morphology and JC-1 in HK-2 cells

HK-2 cells were incubated in DMEM/F12 medium with or without 80 mg I/mL of iohexol at 37°C for 2 h. HK-2 cell mitochondria were labeled with MitoTracker Red CMXRos probe (75 nmol/L, # M75112, Invitrogen, Carlsbad, USA) and photographed with a laser confocal microscope (Olympus, Japan). To determine MMP levels, HK-2 cells were treated with the JC-1 probe. The JC-1 staining working solution (#C2006, Beyotime, Jiangsu, China) was incubated with HK-2 cells at 37°C for 20 min, followed by imaging using a laser confocal microscope (Olympus, Japan). Green fluorescence corresponds to JC‑1 monomers, whereas red fluorescence indicates JC‑1 aggregates. MMP levels were evaluated by calculating the fluorescence ratio of JC‑1 aggregates (red) to monomers (green).

### 2.6. Cell apoptosis assay

The proportion of apoptotic cells was detected using the apoptosis kit (# C1062M, Beyotech, China). HK-2 cells were harvested by 0.25% trypsin without EDTA (# 60-00-4, MCE, USA) after iohexol treatment for 2 h. Cells were washed three times with PBS and resuspended in 100 μL of 1 × Binding Buffer. Then, cells were stained with 5 μL Annexin V-FITC and 5 μL of PI for 10 min in the dark. Finally, stained cells were assessed by flow cytometry (Beckman Coulter, Brea, CA, USA).

### 2.7. Western blotting

Total protein from HK-2 cells was obtained with RIPA lysis buffer containing 1% phenylmethylsulfonyl fluoride (PMSF) (# P0013D, Beyotime, China). Protein concentration was detected using the BCA Protein Assay Kit (# E112-01, Vazyme Biotech, China). A total of 25−50 µg of proteins was separated by SDS-polyacrylamide electrophoresis gels. Proteins were then transferred to a PVDF membrane. The membranes were blocked with 5% skimmed milk powder for 1 h at room temperature and then incubated with specific primary antibodies. After overnight incubation at 4°C, the membranes were rinsed four times with TBST and then incubated with secondary antibodies for 1 h at room temperature. Finally, the bands were visualized by enhanced chemiluminescence (# E423-01, Vazyme Biotech, China). The intensity was quantified using ImageJ software. Information on the antibodies used is detailed in Supplementary Information S1 Table.

### 2.8. Statistical analyses

Statistical analyses were carried out using GraphPad Prism 8 and SPSS Statistics v23.0. Data were presented as mean ± standard deviation (SD). Comparisons between two groups were performed using paired or unpaired t-tests. Multiple comparisons were performed using one-way ANOVA with Tukey’s post hoc test. *P* < 0.05 was regarded as statistically significant. All experiments were repeated three times independently.

## 3. Results

### 3.1. Iohexol induces cytotoxicity in HK-2 cells

Some studies have shown that iohexol induces a more pronounced decrease in HK-2 cells viability than iodixanol [25]. Therefore, iohexol was selected to establish the in-vitro injury model in HK-2 cells. To verify the cytotoxicity effect and optimal modeling dose of iohexol on HK-2 cells, the viability of HK-2 cells treated with different concentrations of iohexol was detected by the CCK-8 assay. Our results demonstrated that iohexol caused a concentration-dependent decrease in cell viability, and a concentration of 80 mg I/mL of iohexol could decrease the cell viability of HK-2 cells to 63.44% (supplementary information, S1 Fig). Thus, we selected 80 mg I/mL of iohexol for 2 h on HK-2 cells as the CA-AKI group for subsequent studies.

### 3.2. mRNA sequencing and data analysis

**Fig 1:**
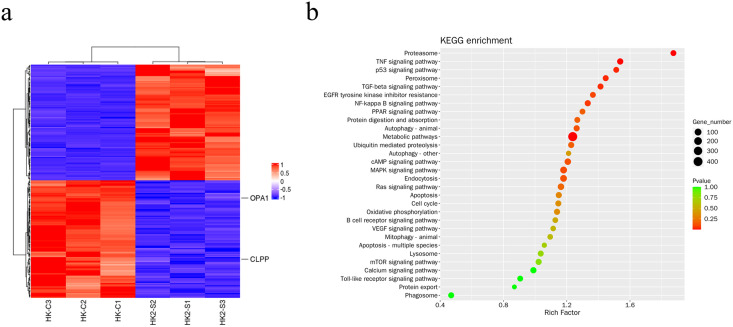
Global transcriptional changes in HK-2 cells with or without iohexol treatment were examined by mRNA-seq. **(a)** Heatmap of dysregulated genes in HK-2 cells treated with or without iohexol. n = 3. **(b)** KEGG enrichment scatterplot of differentially expressed genes in HK-2 cells treated with or without iohexol.

We further investigated the global transcriptional changes in HK-2 cells with or without iohexol treatment by mRNA sequencing (mRNA-seq). Iohexol treatment led to the dysregulation of hundreds of genes in HK-2 cells, among which *OPA1* and *ClpP* were significantly upregulated (Fig 1a). Furthermore, KEGG pathway analysis showed that proteasome-related signaling was significantly altered in iohexol-induced HK-2 cell injury (Fig 1b). GO biological process analysis revealed that the dysregulated genes were associated with mitochondrial dynamics-related processes and proteasome-related biological functions in iohexol-treated HK-2 cells (supplementary information, S2 Fig). These transcriptomic findings suggested that ClpP and mitochondrial dynamics-related pathways may be involved in the early cellular response to iohexol exposure.

### 3.3. Iohexol induced mitochondrial morphological alterations and MMP loss in HK-2 cells

**Fig 2:**
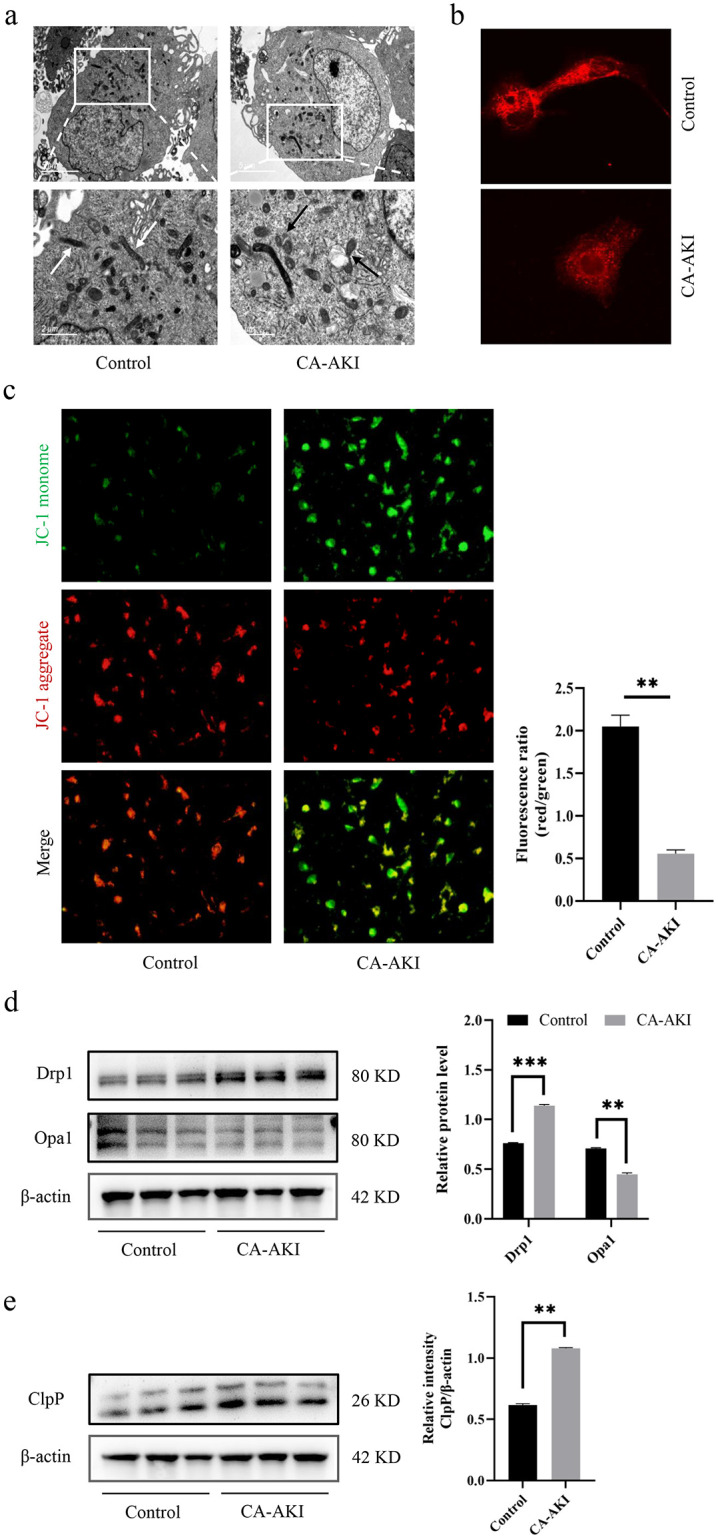
Effect of iohexol exposure on mitochondrial morphology, MMP, and mitochondrial dynamics-related proteins in HK-2 cells. **(a)** Representative transmission electron microscopy images of mitochondria in HK-2 cells treated with or without iohexol (magnification × 5000, scale bar 5 μm; × 12,000, scale bar 2 μm). White arrows indicate relatively elongated mitochondria with preserved ultrastructure. Black arrows indicate fragmented mitochondria (upper) and severely damaged mitochondria with vacuolization (lower). **(b)** Mitochondria were stained with MitoTracker Red CMXRos, and changes in mitochondrial morphology were observed in different groups (original magnification ×1,000). **(c)** MMP levels were assessed in each group by JC-1 staining. The red‑to‑green fluorescence ratio reflects relative MMP levels. Scale bar, 50 μm. **(d)** The expression of Drp1 and Opa1 proteins was detected by Western blotting. **(e)** The expression of ClpP protein was detected by Western blotting. n = 3, data are expressed as mean ± SD. ** *P < 0.01*, *** *P < 0.001*.

It has been shown that disorders in mitochondrial dynamics play a crucial role in the occurrence and progression of contrast-induced nephropathy [26]. We observed changes in mitochondrial morphology in HK-2 cells with and without iohexol treatment by transmission electron microscopy (TEM). Our data revealed that HK-2 cells treated with iohexol exhibited marked mitochondrial morphological damage, characterized by prominent mitochondrial fragmentation, cristae disruption, and vacuolization (Fig 2a). MitoTracker Red staining further revealed that the mitochondria in iohexol-treated HK-2 cells became shortened and appeared more fragmented, suggesting disruption of the mitochondrial network and mitochondrial fission-related morphological changes (Fig 2b). We additionally evaluated MMP levels, an early marker of mitochondrial injury, using JC‑1 staining. A significant reduction in MMP levels was observed in iohexol‑treated HK‑2 cells (Fig 2c). Collectively, these findings suggested that iohexol triggers early mitochondrial injury in HK‑2 cells, as reflected by altered mitochondrial morphology and reduced MMP, rather than comprehensive impairment of overall mitochondrial function.

Subsequently, the expression of Drp1 and Opa1 proteins was detected by Western blotting. Compared with the control group, the expression of the mitochondrial fission-related protein Drp1 was significantly increased, whereas the expression of the mitochondrial fusion protein Opa1 was significantly decreased in the CA-AKI group (Fig 2d). These results suggest that iohexol exposure is associated with increased mitochondrial fission-related changes and reduced fusion-related protein expression in HK-2 cells. In addition, we examined the expression of mitochondrial caseinolytic peptidase P (ClpP) in the CA-AKI group. The CA-AKI group demonstrated a significant increase in ClpP protein levels compared to the control group (Fig 2e).

### 3.4. Apoptosis increase is caused by iohexol treatments in HK-2 cells

**Fig 3:**
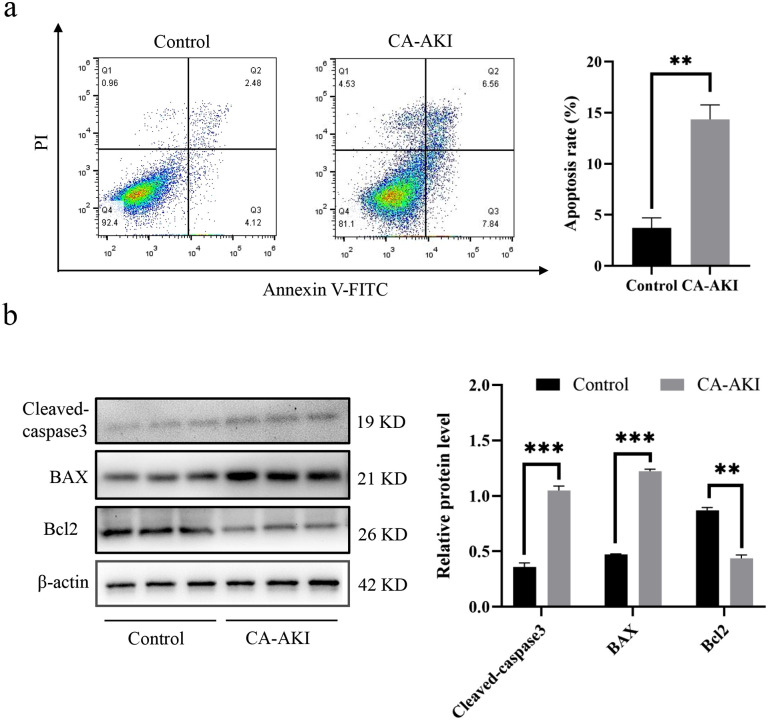
Effect of iohexol exposure on apoptosis in HK-2 cells. **(a)** Iohexol-induced HK-2 cell apoptosis was assessed by flow cytometry. The Q2 and Q3 quadrants represent apoptotic cells. **(b)** Western blotting was used to detect the protein levels of cleaved-caspase 3, BAX, and Bcl-2 in HK-2 cells. n = 3, data are expressed as mean ± SD. ** *P < 0.01*, *** *P < 0.001*.

Based on the fact that mitochondrial injury often leads to apoptosis [27], we also assessed apoptosis in HK-2 cells. Compared with the control group, the CA-AKI group showed increased apoptosis as assessed by Annexin V staining and flow cytometry (2.05% vs. 14.36%, *P* < 0.01) (Fig 3a). Furthermore, the increased apoptosis in the CA-AKI group was further supported by upregulated levels of the apoptosis-related proteins cleaved-caspase 3 and Bax, as well as downregulated levels of the anti-apoptotic protein Bcl-2 (Fig 3b).

### 3.5. Effect of ClpP knockdown on cell viability in iohexol-treated HK-2 cells

In iohexol-treated HK-2 cells, based on the results of mRNA sequencing and ClpP protein expression, LV-ClpP-shRNA was used to knockdown ClpP and then explore the role of ClpP in HK-2 cells. The knockdown efficiency of ClpP in HK-2 cells was analyzed using qPCR and Western blotting (supplementary information, S3 Fig). The control shRNA (Scramble) did not affect the cell viability of HK-2 cells compared with normal cultured HK-2 cells (supplementary information, S4a Fig). Compared with the control group, ClpP knockdown significantly increased the cell viability of iohexol-treated HK-2 cells (supplementary information, S4b Fig).

### 3.6. Knockdown of ClpP with LV-ClpP-shRNA increased the expression of Opa1 protein in iohexol-treated HK-2 cells

**Fig 4:**
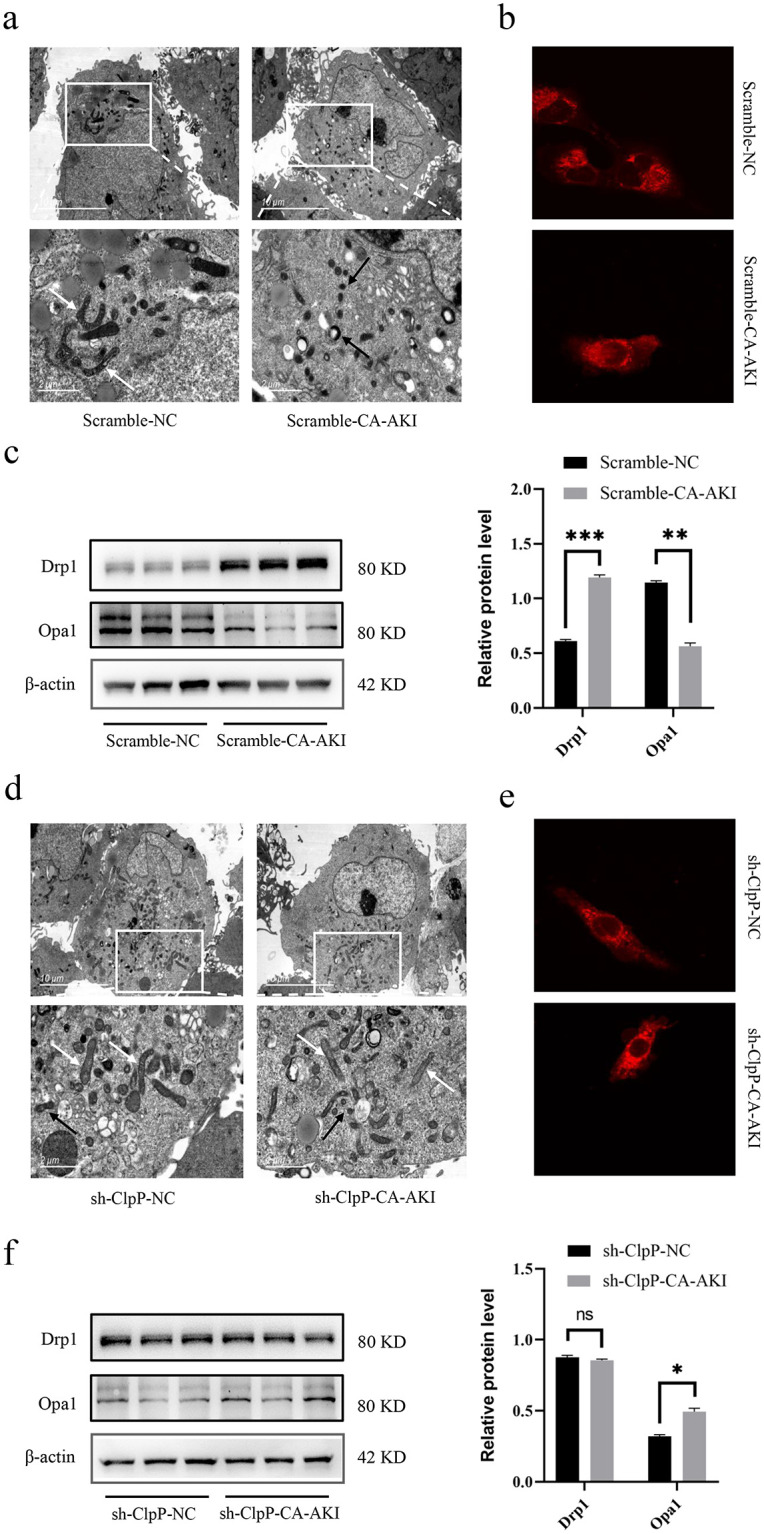
(a, d) Mitochondrial morphological changes were observed after knockdown of ClpP in HK-2 cells. White arrows indicate relatively elongated mitochondria; black arrows indicate fragmented or damaged mitochondria. (b, e) Mitochondria were stained with MitoTracker Red CMXRos, and changes in mitochondrial morphology were observed in the different groups (original magnification ×1,000). (c, f) Western blotting was used to detect the protein levels of Drp1 and Opa1 after knockdown of ClpP in HK-2 cells. NC, negative control. n = 3, data are expressed as mean ± SD. *N.S.* no significance. * *P < 0.05*, ** *P < 0.01*, *** *P < 0.001*.

We investigated the effect of ClpP knockdown on iohexol-induced mitochondrial morphological changes in HK-2 cells. As shown in Fig 4a, the control shRNA (Scramble) did not reverse iohexol‑triggered mitochondrial structural damage, whereas ClpP knockdown partially attenuated these morphological abnormalities (Fig 4d). Consistent with these findings, MitoTracker Red staining revealed that the control shRNA (Scramble) failed to attenuate iohexol‑induced mitochondrial shrinkage and fragmentation (Fig 4b), while ClpP knockdown partially preserved mitochondrial network morphology and reduced mitochondrial fragmentation-related changes (Fig 4e). Western blot analysis was performed to detect the expression of the mitochondrial fission‑associated protein Drp1 and fusion‑associated protein Opa1. The control shRNA (Scramble) did not affect the expression trends of Drp1 and Opa1 protein (Fig 4c). Notably, ClpP knockdown significantly increased the expression of Opa1 protein. However, the expression of Drp1 protein had no significant change in iohexol-treated HK-2 cells (Fig 4f). Taken together, these results suggest that ClpP deficiency may partially preserve mitochondrial homeostasis in iohexol-injured HK-2 cells, mainly in association with increased OPA1 expression and improved mitochondrial morphology, rather than through significant changes in Drp1 expression.

**Fig 5:**
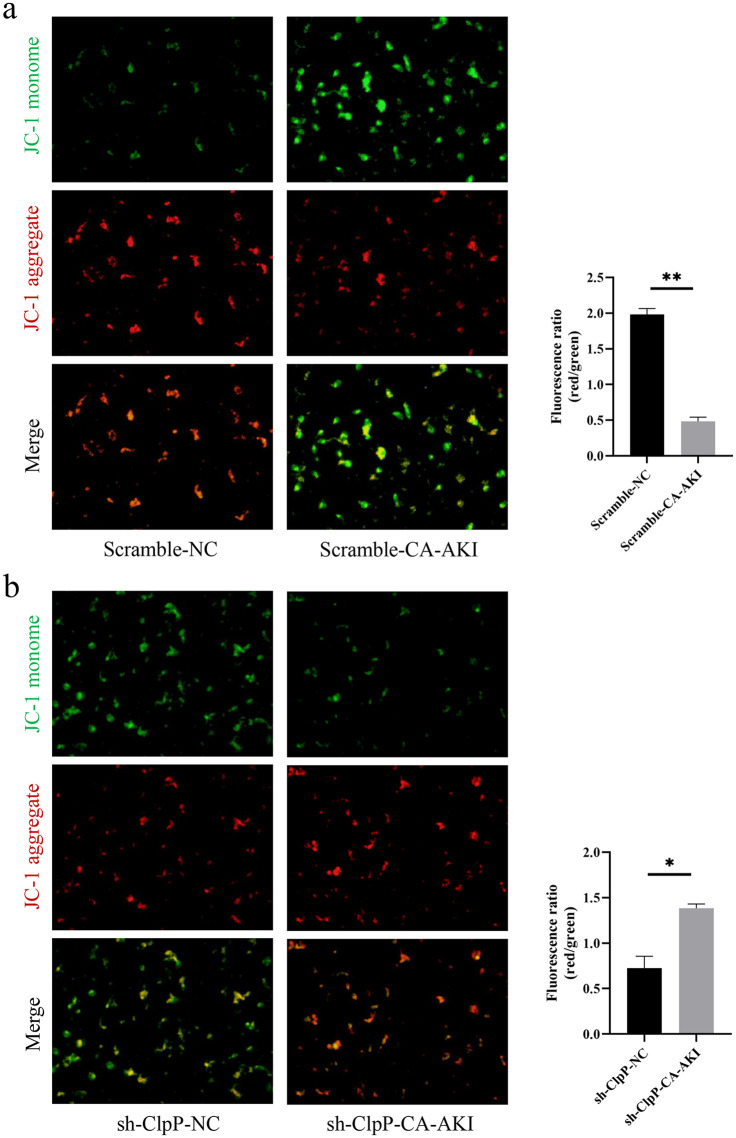
(a, b) MMP levels were assessed in each group by JC-1 staining. The red‑to‑green fluorescence ratio reflects relative MMP levels. Scale bar, 50 μm. n = 3 per group. Data are expressed as mean ± SD.

To further characterize how ClpP deficiency is associated with early mitochondrial injury, we assessed MMP levels in iohexol-treated HK-2 cells using JC-1 staining. The control shRNA (Scramble) did not alter the trend of MMP changes in HK-2 cells (Fig 5a). Notably, ClpP knockdown significantly restored MMP levels and reversed iohexol‑triggered mitochondrial membrane potential decline (Fig 5b). These results indicate that ClpP silencing may mitigate early mitochondrial injury by preserving MMP in iohexol‑exposed HK‑2 cells, rather than fully rescuing overall mitochondrial function.

### 3.7. Knockdown of ClpP with LV-ClpP-shRNA decreased the percentage of apoptotic cells in iohexol-treated HK-2 cells

**Fig 6:**
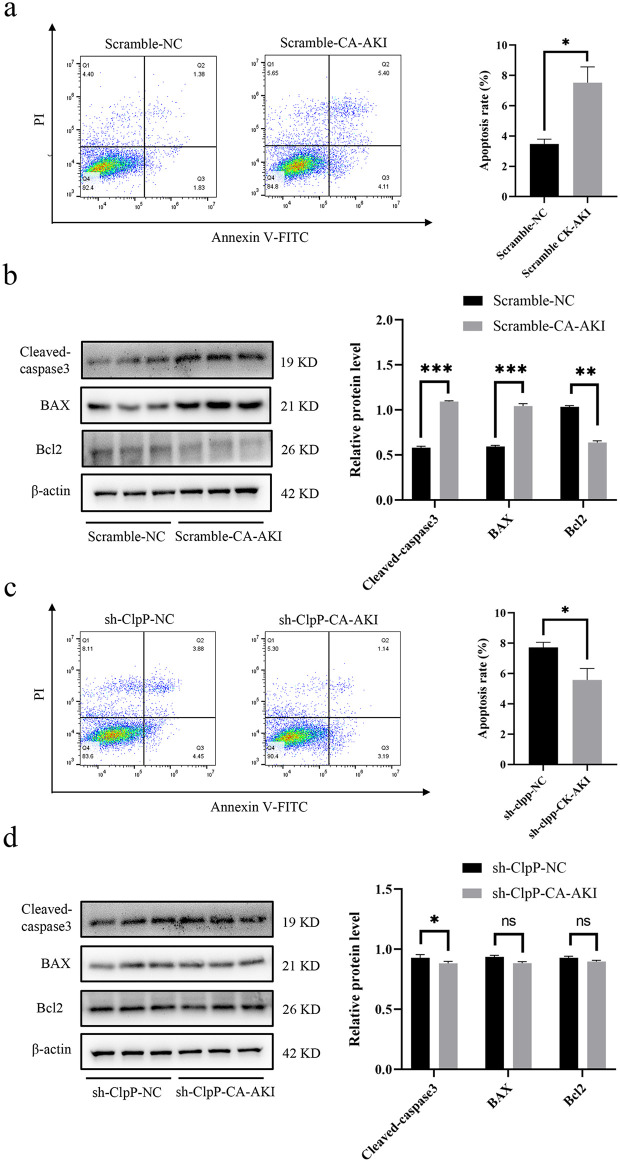
Effect of knockdown ClpP on the level of apoptosis in iohexol-induced HK-2 cells. **(a, c)** The percentage of apoptotic cells was assessed by flow cytometry in different groups. The Q2 and Q3 quadrants represent apoptotic cells. **(b, d)** Western blotting was used to detect the protein levels of cleaved-caspase 3, BAX, and Bcl-2 in different groups. NC, negative control. n = 3, data are expressed as mean ± SD. *N.S.* no significance. * *P < 0.05*, ** *P < 0.01*, *** *P < 0.001*.

Subsequently, we also investigated the effect of knocking down ClpP on apoptosis in iohexol-treated HK-2 cells. As shown in Fig 6a, b, the control shRNA (Scramble) did not affect the trend of apoptosis in HK-2 cells. Flow cytometry analysis indicated that ClpP knockdown attenuated the increase of iohexol-induced apoptosis in HK-2 cells (7.73% vs. 5.58%, *P* < 0.05) (Fig 6c). ClpP deficiency also reduced the levels of iohexol-induced apoptosis-related proteins cleaved-caspase 3, and Bax (Fig 6d). Collectively, our results suggested that ClpP knockdown may inhibit the increase in apoptosis in iohexol-treated HK-2 cells.

## 4. Discussion

Mitochondrial injury is widely considered one of the important contributors to the pathogenesis of CA-AKI [28, 29]. Recent evidence has indicated that ClpP‑mediated mitochondrial quality control is essential for maintaining mitochondrial homeostasis in multiple human diseases [14, 30]. Nevertheless, whether ClpP participates in contrast‑induced mitochondrial injury in renal tubular epithelial cells remains poorly defined. In this study, we found that treatment with 80 mg I/mL iohexol decreased MMP levels, disturbed mitochondrial dynamics balance, and triggered apoptosis in HK-2 cells. We also found that ClpP expression was significantly upregulated in iohexol-treated HK-2 cells. Notably, ClpP knockdown alleviated iohexol‑induced injury, which was accompanied by increased OPA1 expression, partial restoration of MMP, improved mitochondrial ultrastructure, and reduced apoptosis. Collectively, these findings suggest that ClpP deficiency may exert cytoprotective effects against iohexol-induced HK-2 cell injury through changes associated with mitochondrial dynamics, MMP preservation, and apoptosis reduction.

CA-AKI represents a major cause of acute renal dysfunction and mortality among hospitalized patients, severely compromising patient prognosis [31]. Current clinical interventions for CA‑AKI remain limited, hydration therapy with physiological saline as the primary preventive strategy [32]. Mounting evidence highlights that mitochondria are central regulators of tubular cell injury during CA‑AKI progression.

Previous studies have demonstrated that contrast agents filtered by the kidneys are converted into positively charged metabolites, which accumulate on the negatively charged mitochondrial membrane and reduce MMP levels [33]. This leads to drastic changes in mitochondrial dynamics, generating mitochondrial reactive oxygen species and abnormal mitochondrial pathology via restraining ATP production and energy metabolism [33, 34]. Among dynamic changes, excessive mitochondrial fission is increasingly recognized as a critical driver of inflammation amplification and cell death in renal dysfunction [35]. Zhang et al demonstrated that inhibiting Drp1 phosphorylation at Ser616 can attenuate contrast-induced AKI in vitro and in vivo [26]. Meanwhile, Drp1 knockdown in HK-2 cells inhibited cisplatin-induced cell injury and mitochondrial dysfunction [36]. In line with these studies, our results verified that iohexol promoted the expression of Drp1 and inhibited the expression of Opa1 in HK-2 cells. Together with our TEM observations showing mitochondrial fragmentation, cristae disruption, and vacuolization in iohexol-treated cells, these molecular data suggest that dysregulated mitochondrial dynamics may be involved in iohexol-induced mitochondrial morphological damage and tubular epithelial cell injury. Notably, MMP levels measured by JC‑1 staining only reflect mitochondrial membrane depolarization, representing a single early indicator of mitochondrial injury. Our mRNA sequencing analysis further revealed significant alterations in mitochondrial dynamics-related signaling and proteasome‑related pathways upon iohexol exposure. Mitochondrial proteome homeostasis is essential for cellular survival, and disrupted protein quality control leads to proteotoxic stress and subsequent cell death. The cytoplasmic ubiquitin-proteasome system controls the transfer of proteins across the mitochondrial outer membrane and eliminates damaged or misfolded proteins, thereby contributing to mitochondrial homeostasis [37].

ClpP is a protease that specifically controls mitochondrial mass and maintains protein homeostasis mainly by degrading misfolded or damaged proteins in the mitochondrial matrix [38]. Abnormal *ClpP* expression disturbs mitochondrial protein homeostasis and triggers cellular dysfunction in various pathological contexts [39]. In oocytes of *ClpP*^*-/-*^ mouse, ClpP deficiency can cause an imbalance of cellular energy metabolism and disruption of mitochondrial dynamics [40]. Similarly, ClpP silencing induces apoptosis in human ovarian granulosa cells via upregulating BAX and activating caspase 3 [41]. In high-glucose and high-fat treated Min6 cells, knockdown of ClpP exacerbates mitochondrial fission while suppressing mitochondrial fusion [20]. Paradoxically, ClpP downregulation ameliorates mitochondrial cardiomyopathy and partially rescues impaired respiratory function [42]. These inconsistent findings indicate that the biological role of ClpP is highly context‑dependent and requires further investigation. In our study, we observed that the expression of ClpP was significantly elevated in iohexol-treated HK-2 cells. ClpP knockdown increased MMP levels, upregulated the fusion protein Opa1, and reduced apoptotic cell death. These findings suggested that ClpP may be involved in the regulation of iohexol-induced HK-2 cell injury, and that ClpP downregulation may attenuate tubular cell injury in association with altered mitochondrial dynamics, partial MMP preservation, and reduced apoptosis. Nevertheless, the exact molecular mechanism by which ClpP modulates mitochondrial homeostasis requires further exploration.

Our study still has several inherent limitations that should be acknowledged. First, further in vivo studies are warranted to validate the findings obtained from this in vitro HK-2 cell model. Second, although our results suggest that ClpP may be associated with changes in mitochondrial fission-fusion dynamics, the direct molecular interactions between ClpP and the mitochondrial dynamics machinery were not fully elucidated in the present work. Third, as MMP only reflects changes in mitochondrial membrane potential, future multidimensional evaluations of mitochondrial function, such as oxygen consumption rate, ATP production, mitochondrial ROS generation, and respiratory chain activity, are needed to further extend our findings. In addition, TEM provides static ultrastructural evidence but does not directly capture real-time mitochondrial fusion or fission events; therefore, live-cell imaging or quantitative mitochondrial network analysis would be needed in future studies to further validate mitochondrial dynamics. Finally, studies focusing on ClpP in CA‑AKI are still limited, and more mechanistic research is required to uncover the detailed regulatory network of ClpP in contrast‑induced renal injury.

In summary, our study provides preliminary evidence that the mitochondrial protease ClpP is upregulated in iohexol-exposed renal tubular epithelial cells. ClpP knockdown may attenuate iohexol-induced HK-2 cell injury, at least partly through changes associated with mitochondrial dynamics, partial preservation of mitochondrial ultrastructure and MMP, and reduction of apoptosis. These findings suggest that ClpP may represent a potential molecular target for further investigation in CA-AKI. Future in vivo and mechanistic studies are required to clarify the precise role of ClpP-related signaling pathways in contrast-induced renal injury.

## Supporting information

S1 File Full Original Western Blot Images. This file contains all uncropped raw Western blot images underlying the present study.
(PDF)

S1 Fig The HK-2 cells were subjected to different concentrations of iohexol for 2 hours. Cell viability was assessed by the CCK-8 assay. Iohexol at different concentrations (mg I/ml) caused decreased cell viability in a concentration-dependent manner. n = 6 in each group. Data are expressed as mean ± SD. *** *P* < 0.001.
(TIF)

S2 Fig GO-enriched scatterplot of differentially expressed genes in HK-2 cells treated with or without iohexol. (TIF)

S3 Fig The knockdown efficiency of lentiviral transfection was analyzed using Western blot and RT-qPCR. The mRNA and protein expression of *ClpP* in lentiviral-treated HK-2 cells are shown. (a) Representative Western blot analysis of ClpP in HK-2 cells, normalized to β-actin. (b) Relative expression of *ClpP* mRNA levels in HK-2 cells. n = 3 in each group. All data are shown as means ± SEM. ** *P* < 0.01.
(TIF)

S4 Fig (a, b) Cell viability assessed by the CCK8 assay in different groups of HK-2 cells. * *P* < 0.05, ** *P* < 0.01, *** *P* < 0.01.n = 3 in each group. NC, normal control. Data are shown as means ± SD. *N.S.* no significance.
(TIF)

S1 Table The antibodies used for Western blotting (WB). (TIF)
