# Supplementary material for: ClpP deficiency attenuates contrast-induced HK-2 cell injury through changes associated with mitochondrial dynamics and apoptosis
Source: PLoS One. 2026 Jul 2;21(7):e0352422. doi: 10.1371/journal.pone.0352422 (PMC13327117; doi:10.1371/journal.pone.0352422)
Supplement: S1 File — This file contains all uncropped raw Western blot images underlying the present study. (PDF) [file pone.0352422.s001.pdf]

Full original Western blots

HK-2 cells

Figure 3a (n=3)

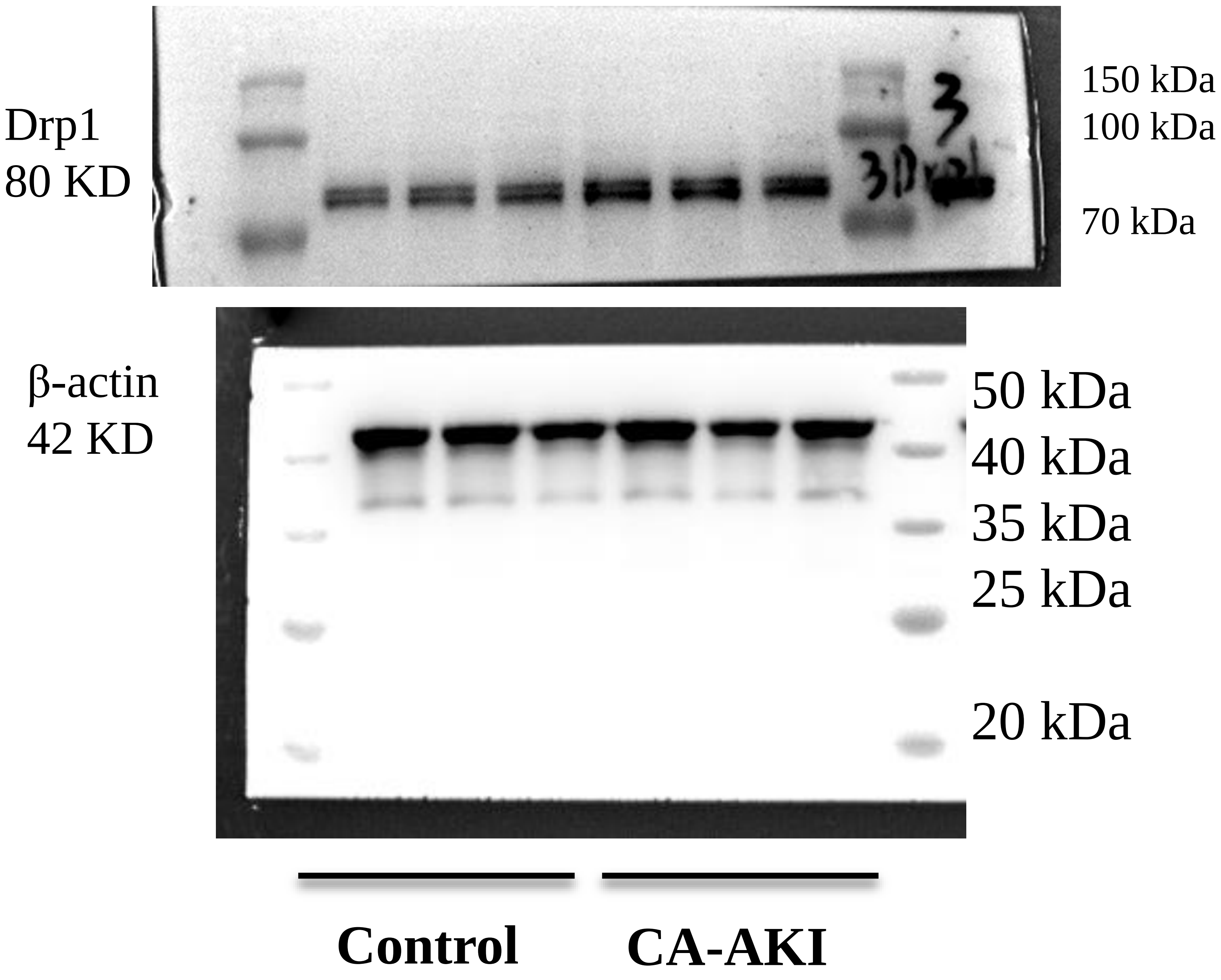

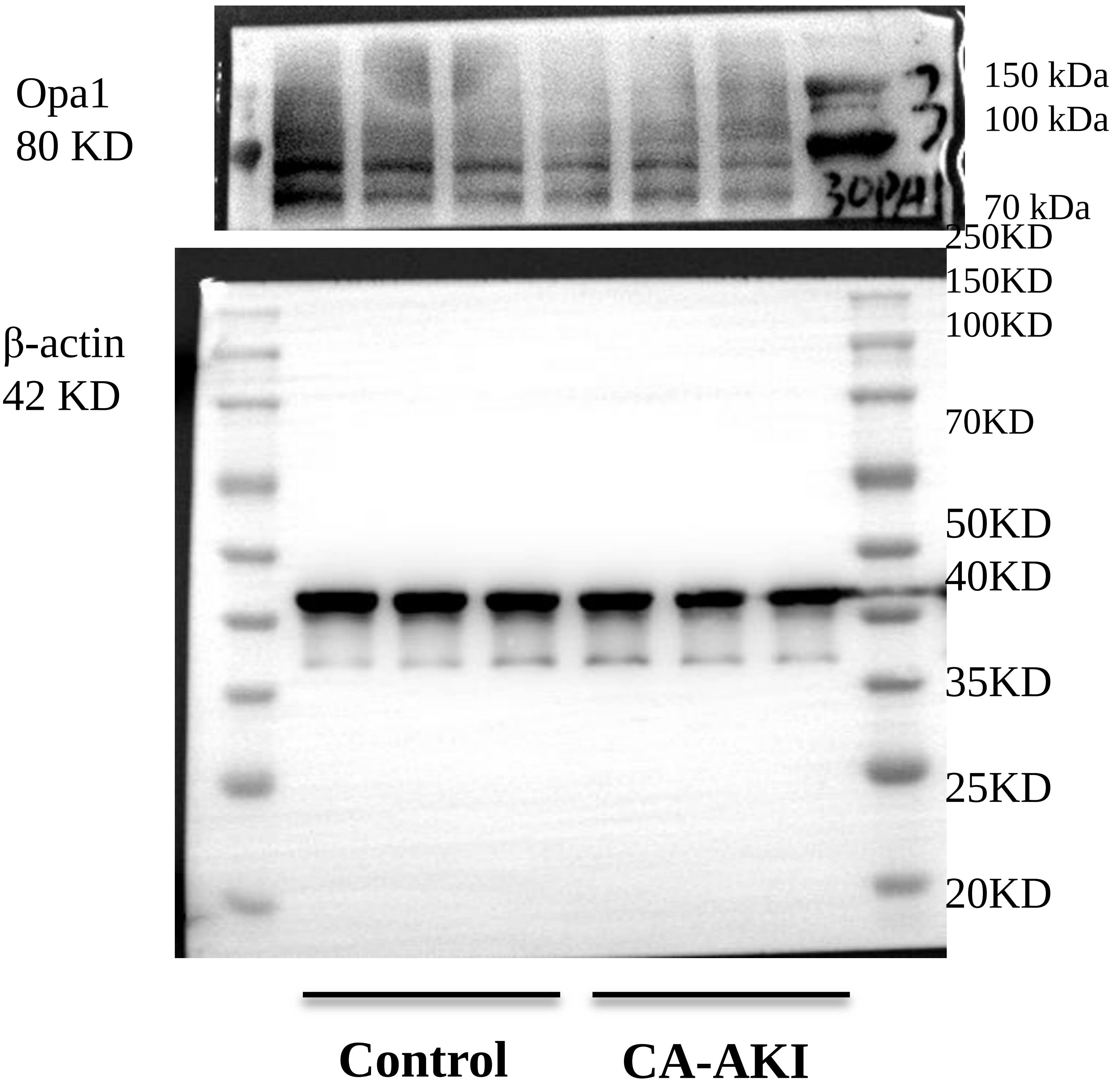

Figure 3b (n=3)

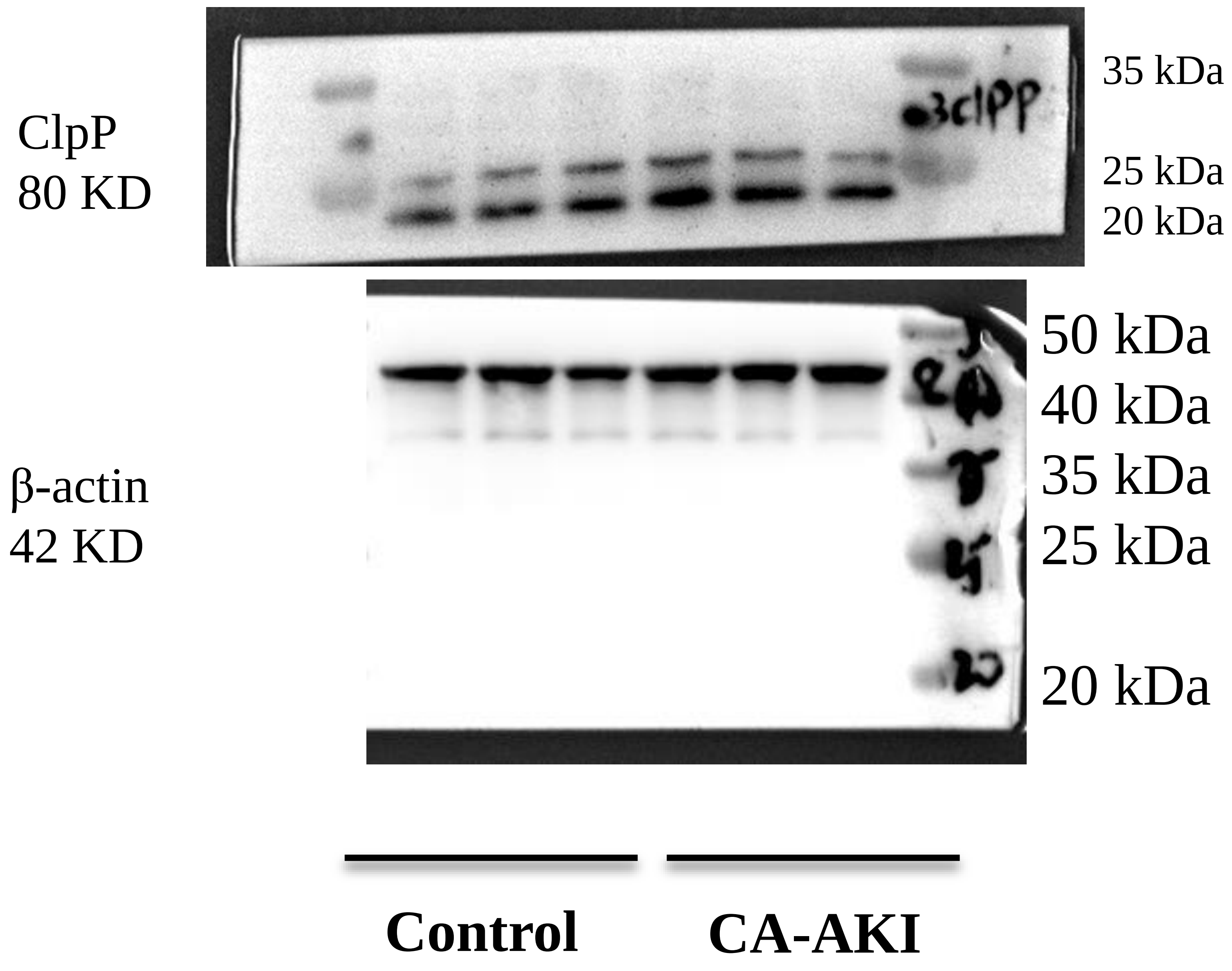
ClpP
80 KD
35 kDa
25 kDa
20 kDa
β-actin
42 KD
50 kDa
40 kDa
35 kDa
25 kDa
20 kDa
Control
CA-AKI

Figure 4b (n=3)

Cleaved-Casepase3
19 KD

β-actin
42 KD

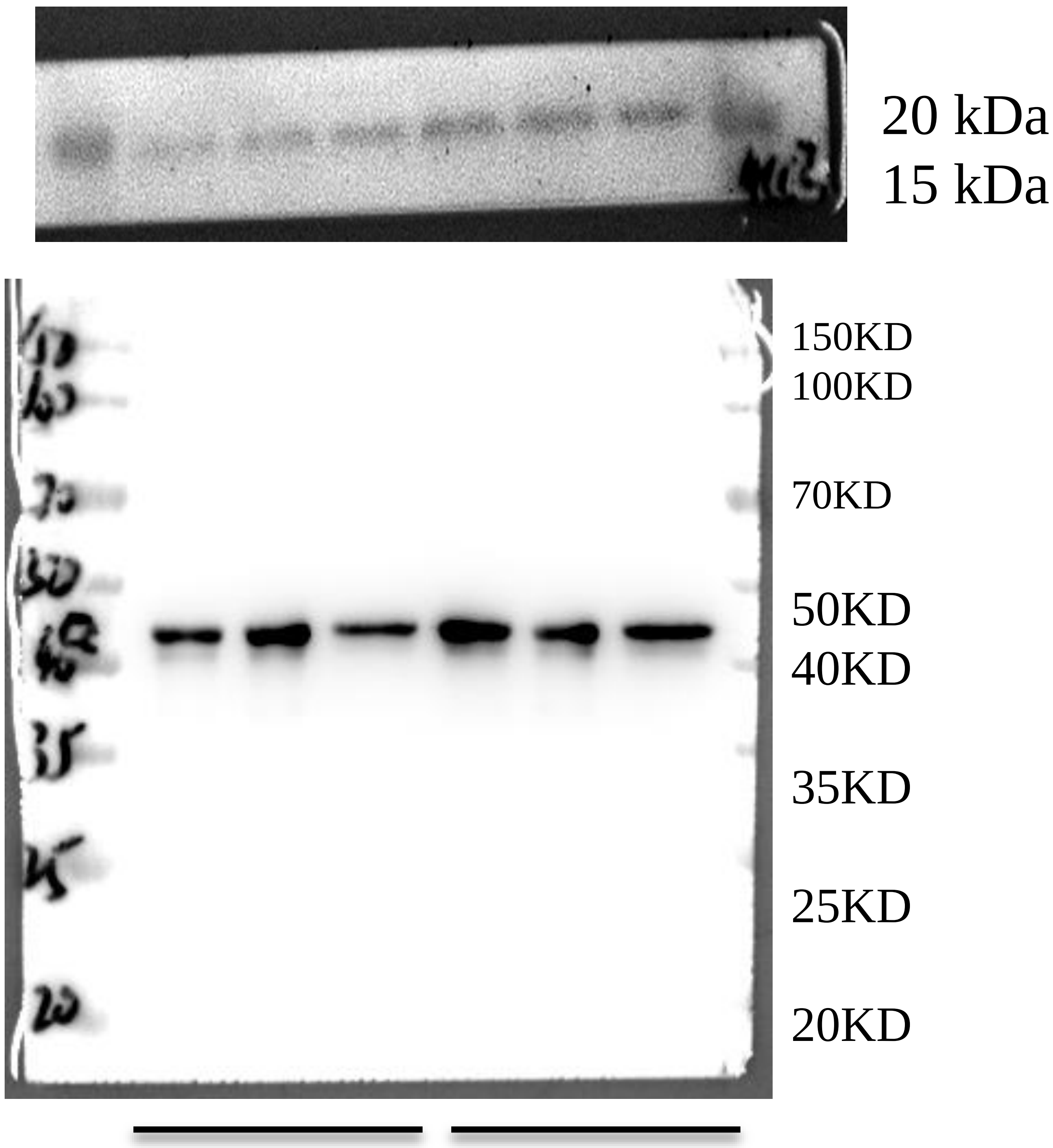

20 kDa
15 kDa

150KD
100KD
70KD
50KD
40KD
35KD
25KD
20KD

Control **CA-AKI**

Caspase3
49 KD

β-actin
42 KD

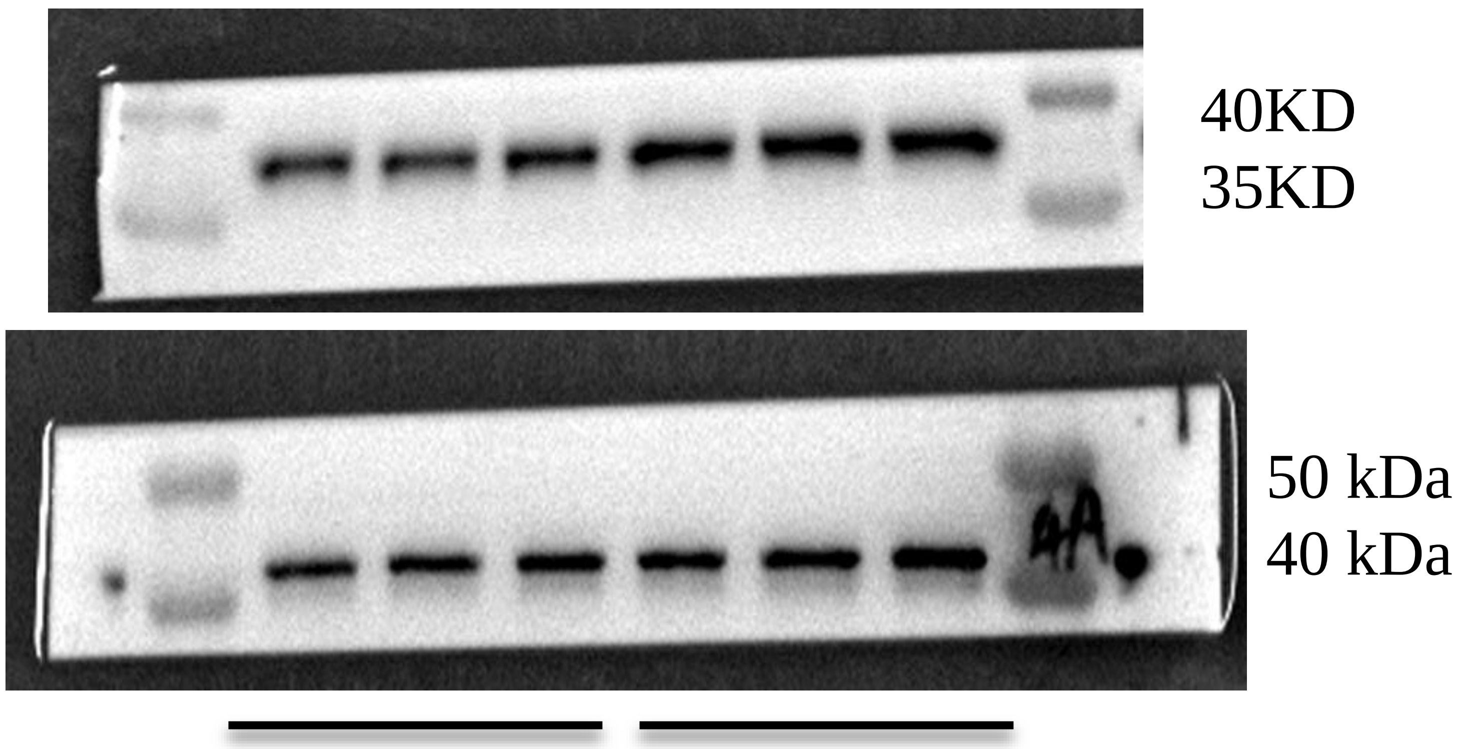

40KD
35KD

50 kDa
40 kDa

Control **CA-AKI**

Figure 4b (n=3)

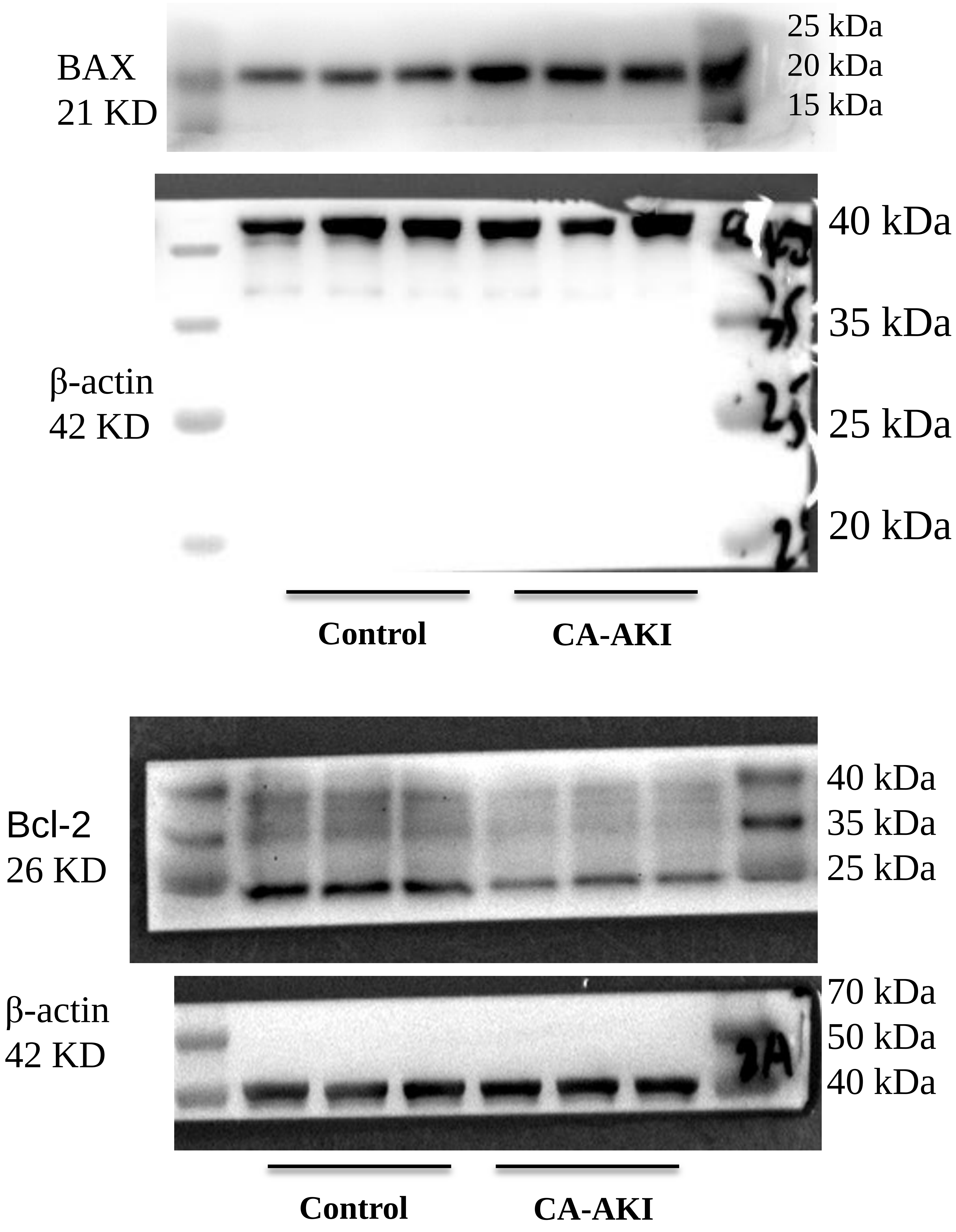
BAX
21 KD
25 kDa
20 kDa
15 kDa
β-actin
42 KD
40 kDa
35 kDa
25 kDa
20 kDa
Control
CA-AKI
Bcl-2
26 KD
40 kDa
35 kDa
25 kDa
β-actin
42 KD
70 kDa
50 kDa
40 kDa
Control
CA-AKI

Figure 5a (n=3)

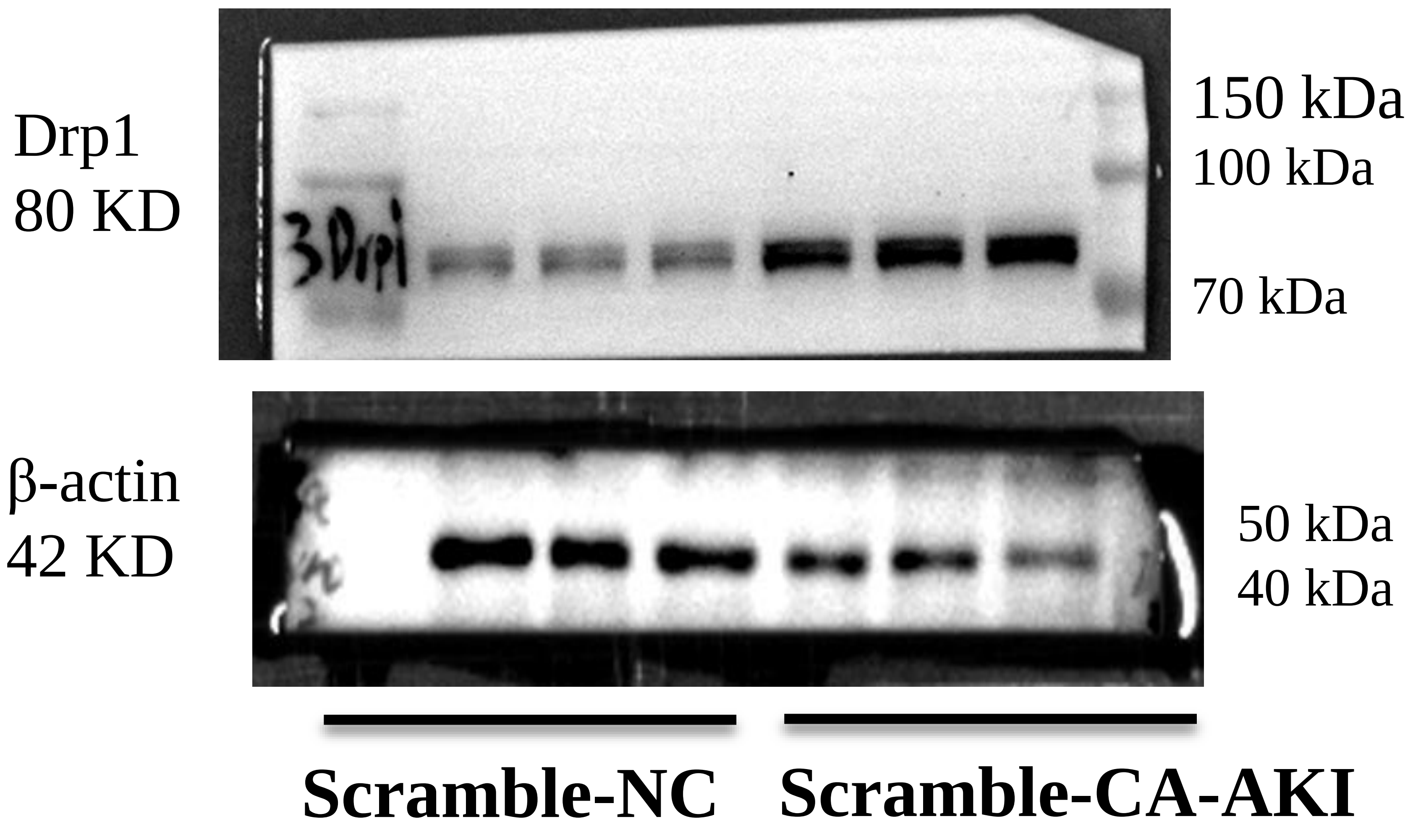
Drp1
80 KD
150 kDa
100 kDa
70 kDa
β-actin
42 KD
50 kDa
40 kDa
Scramble-NC
Scramble-CA-AKI

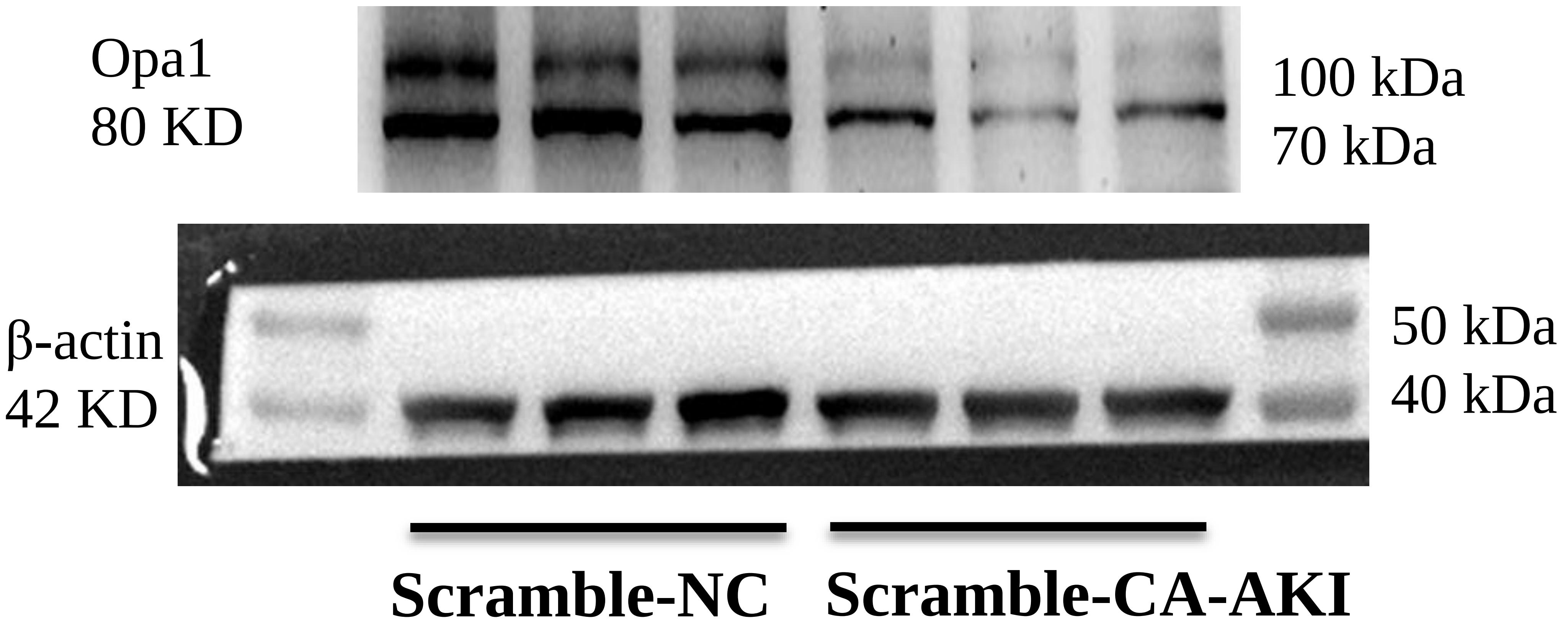
Opa1
80 KD
100 kDa
70 kDa
β-actin
42 KD
50 kDa
40 kDa
Scramble-NC
Scramble-CA-AKI

Figure 5b (n=3)

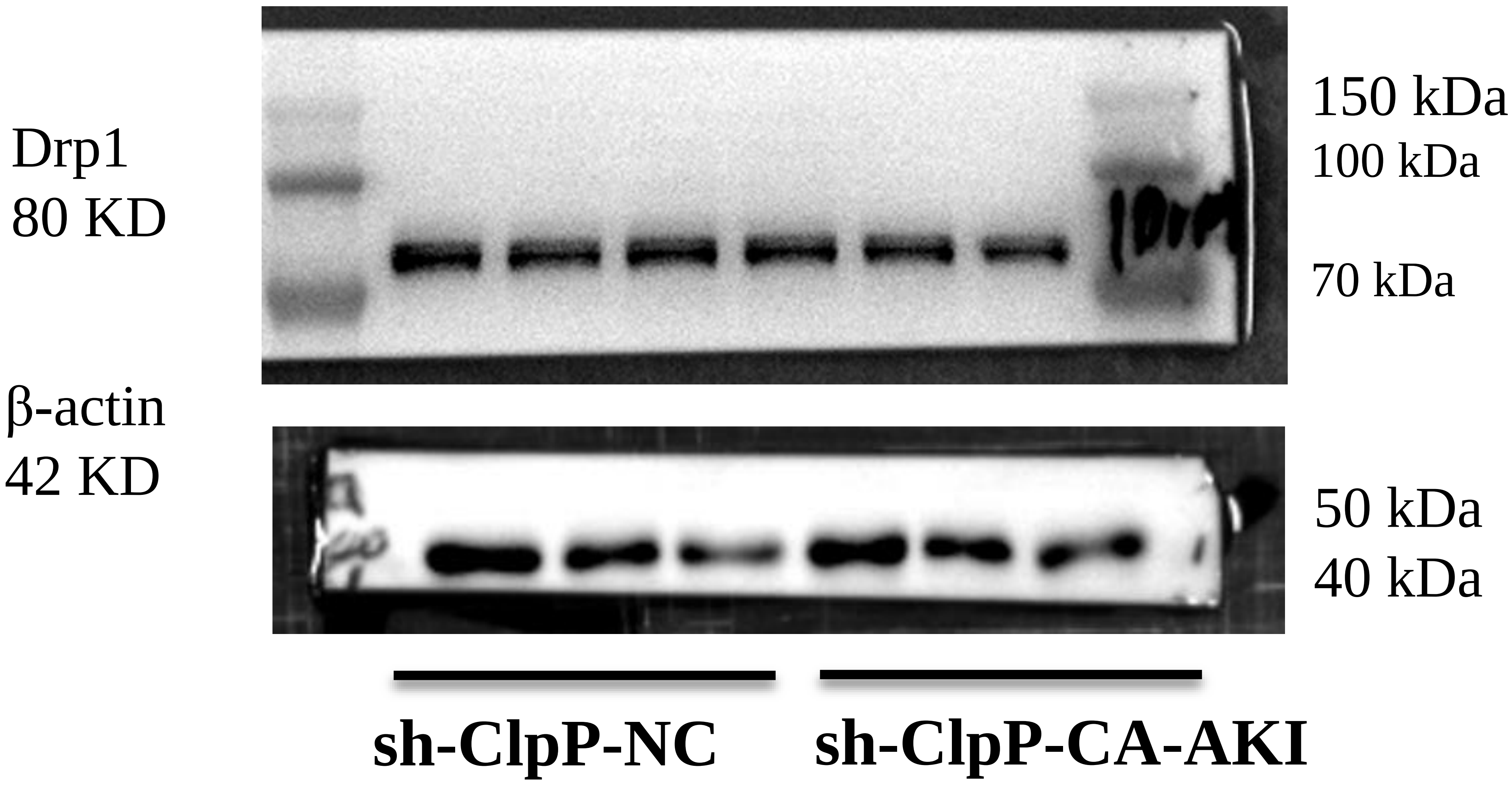
Drp1
80 KD
β-actin
42 KD
150 kDa
100 kDa
70 kDa
50 kDa
40 kDa
sh-ClpP-NC
sh-ClpP-CA-AKI

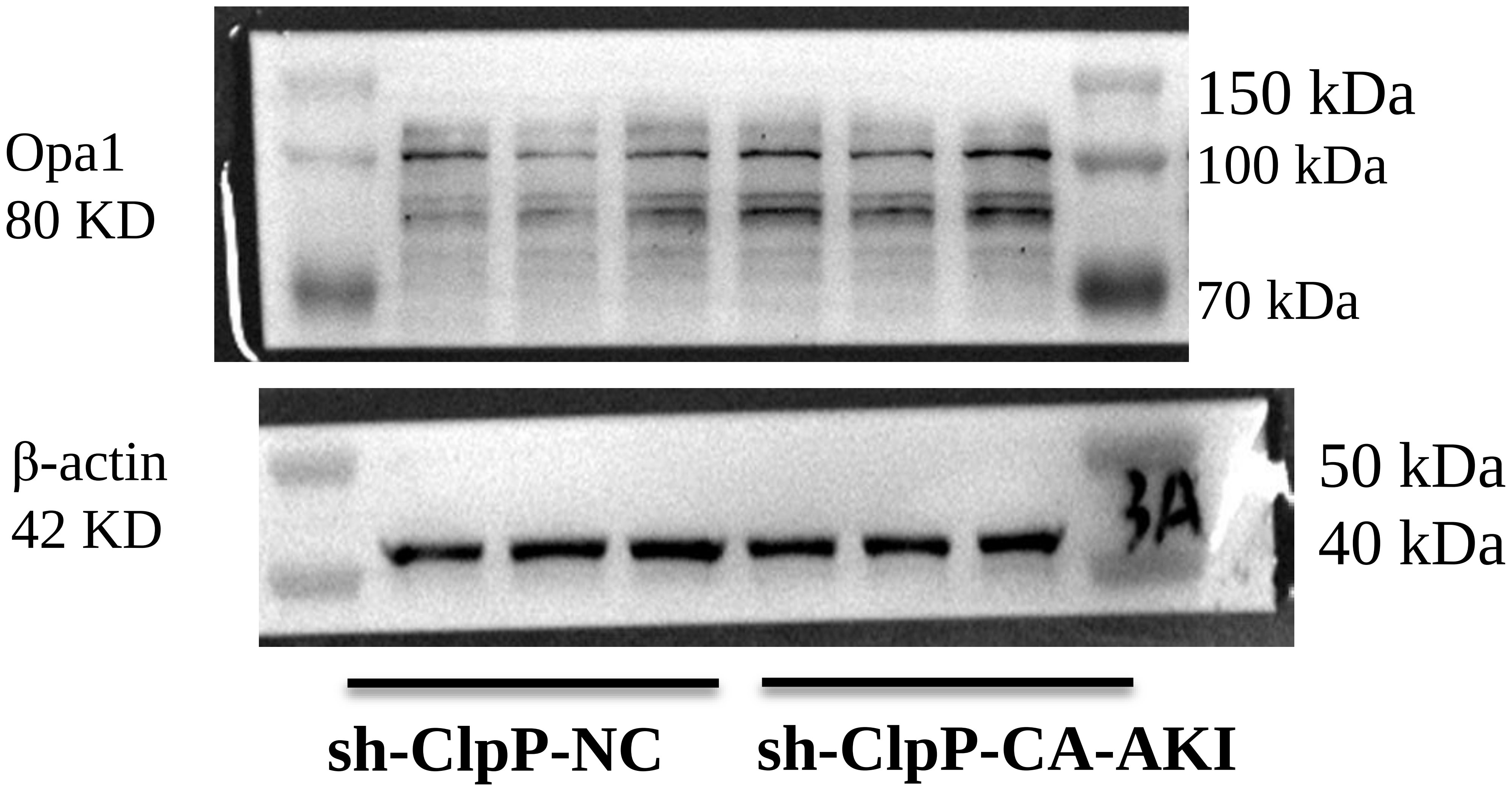
Opa1
80 KD
β-actin
42 KD
150 kDa
100 kDa
70 kDa
50 kDa
40 kDa
sh-ClpP-NC
sh-ClpP-CA-AKI

Figure 7b (n=3)

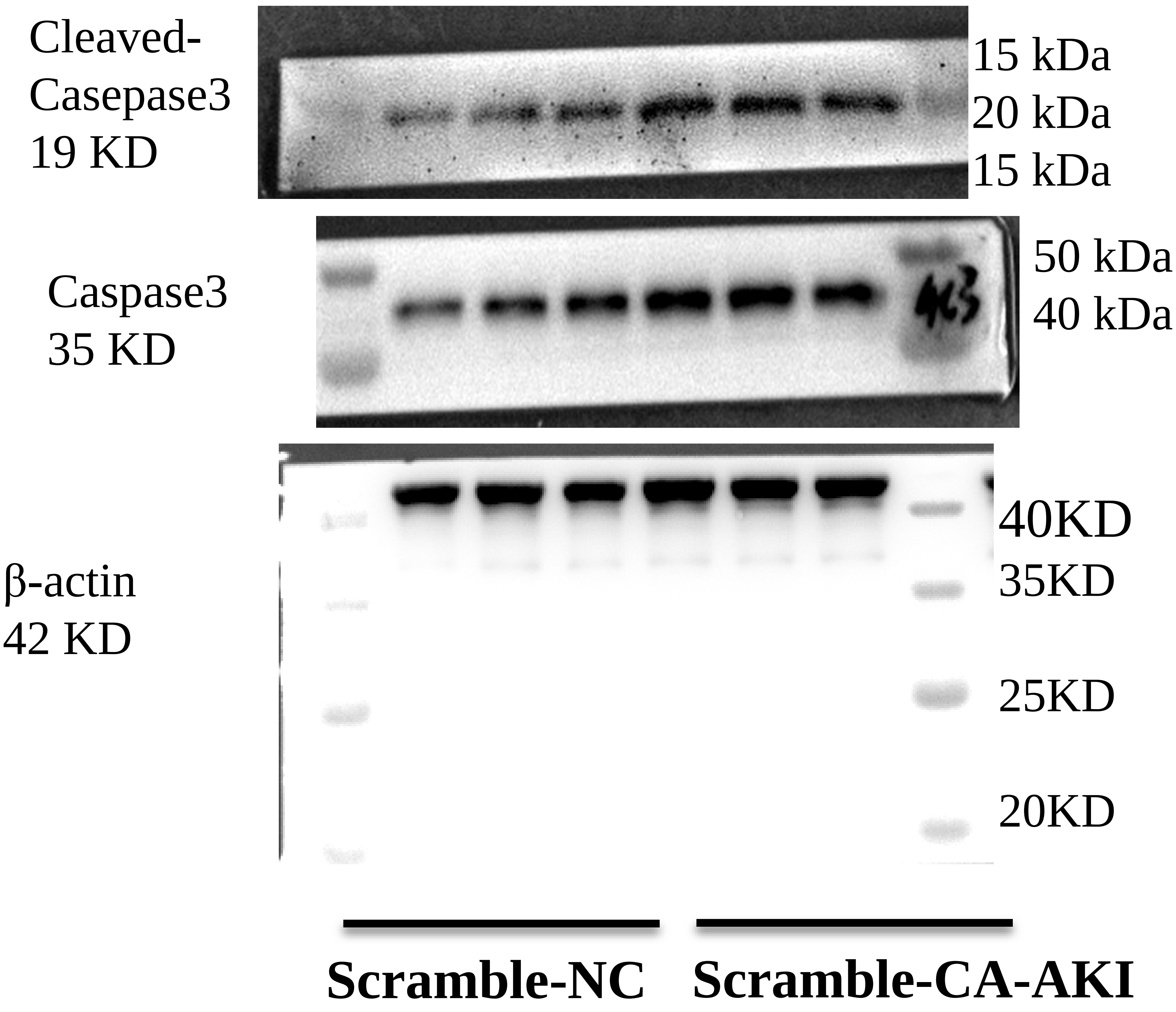
Cleaved-Casepase3
19 KD
15 kDa
20 kDa
15 kDa
Caspase3
35 KD
50 kDa
40 kDa
β-actin
42 KD
40KD
35KD
25KD
20KD
Scramble-NC
Scramble-CA-AKI

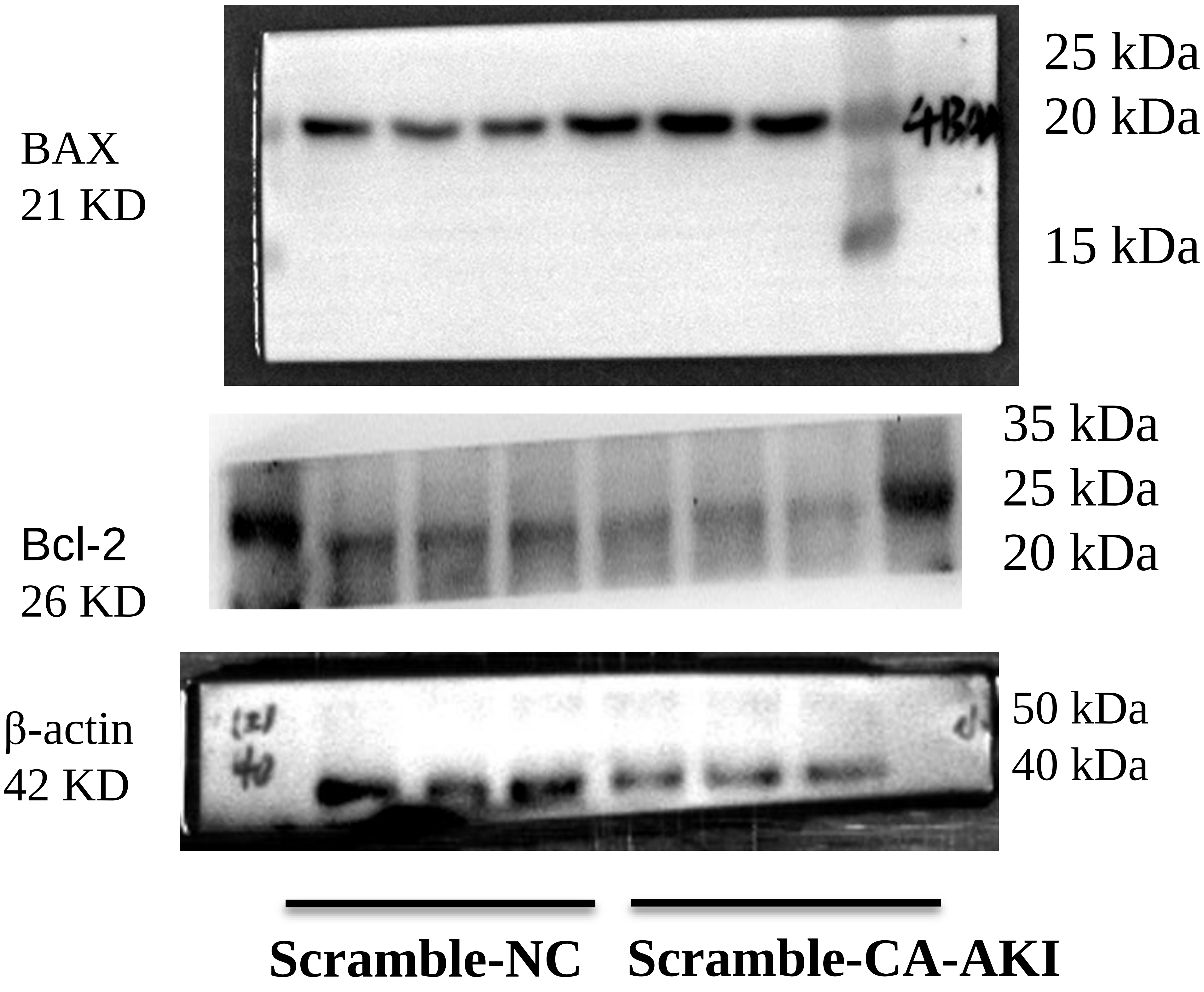
BAX
21 KD
25 kDa
20 kDa
15 kDa
Bcl-2
26 KD
35 kDa
25 kDa
20 kDa
β-actin
42 KD
50 kDa
40 kDa
Scramble-NC
Scramble-CA-AKI

Figure 7d (n=3)

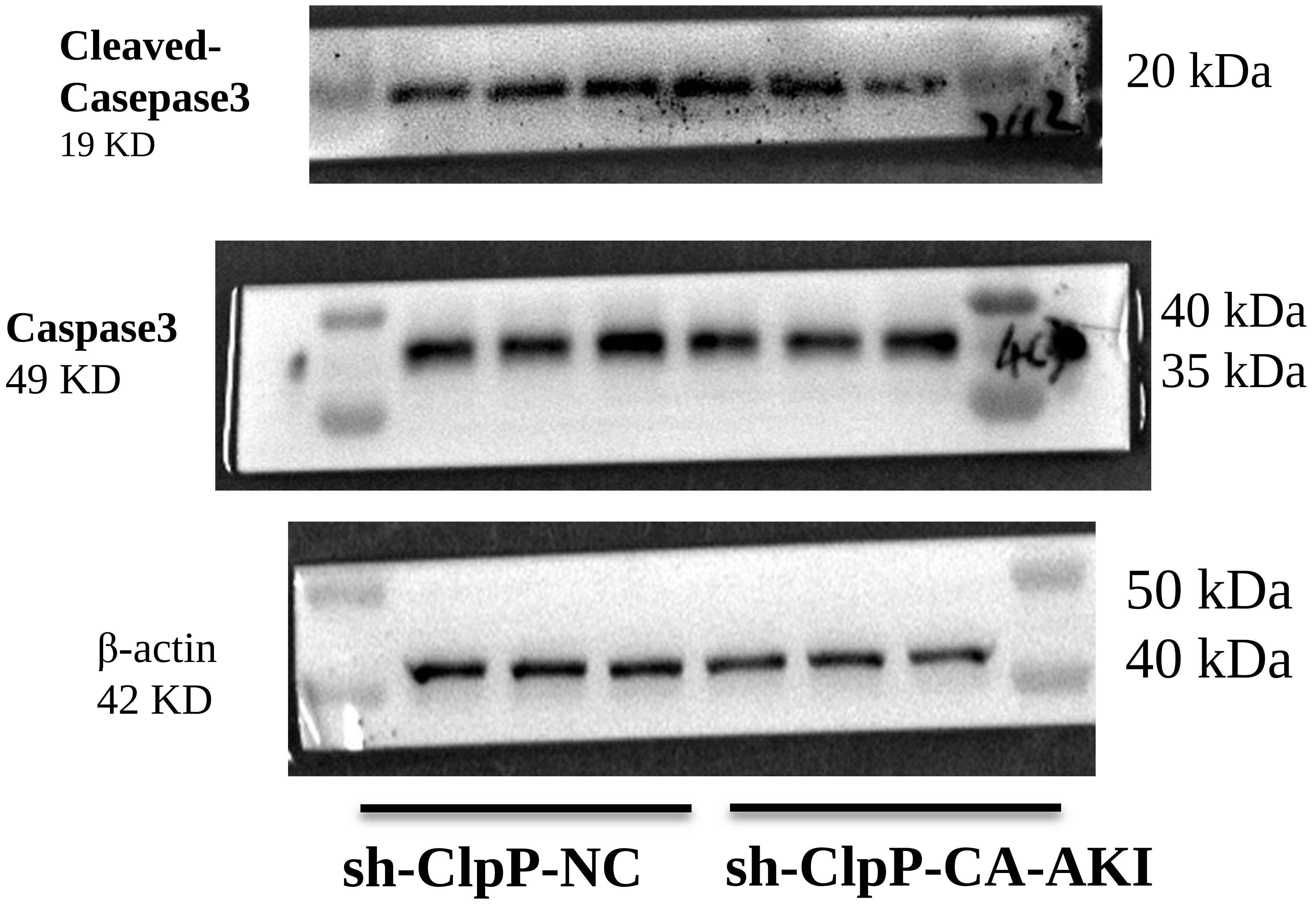
Cleaved-Casepase3
19 KD
20 kDa
Caspase3
49 KD
40 kDa
35 kDa
β-actin
42 KD
50 kDa
40 kDa
sh-ClpP-NC
sh-ClpP-CA-AKI

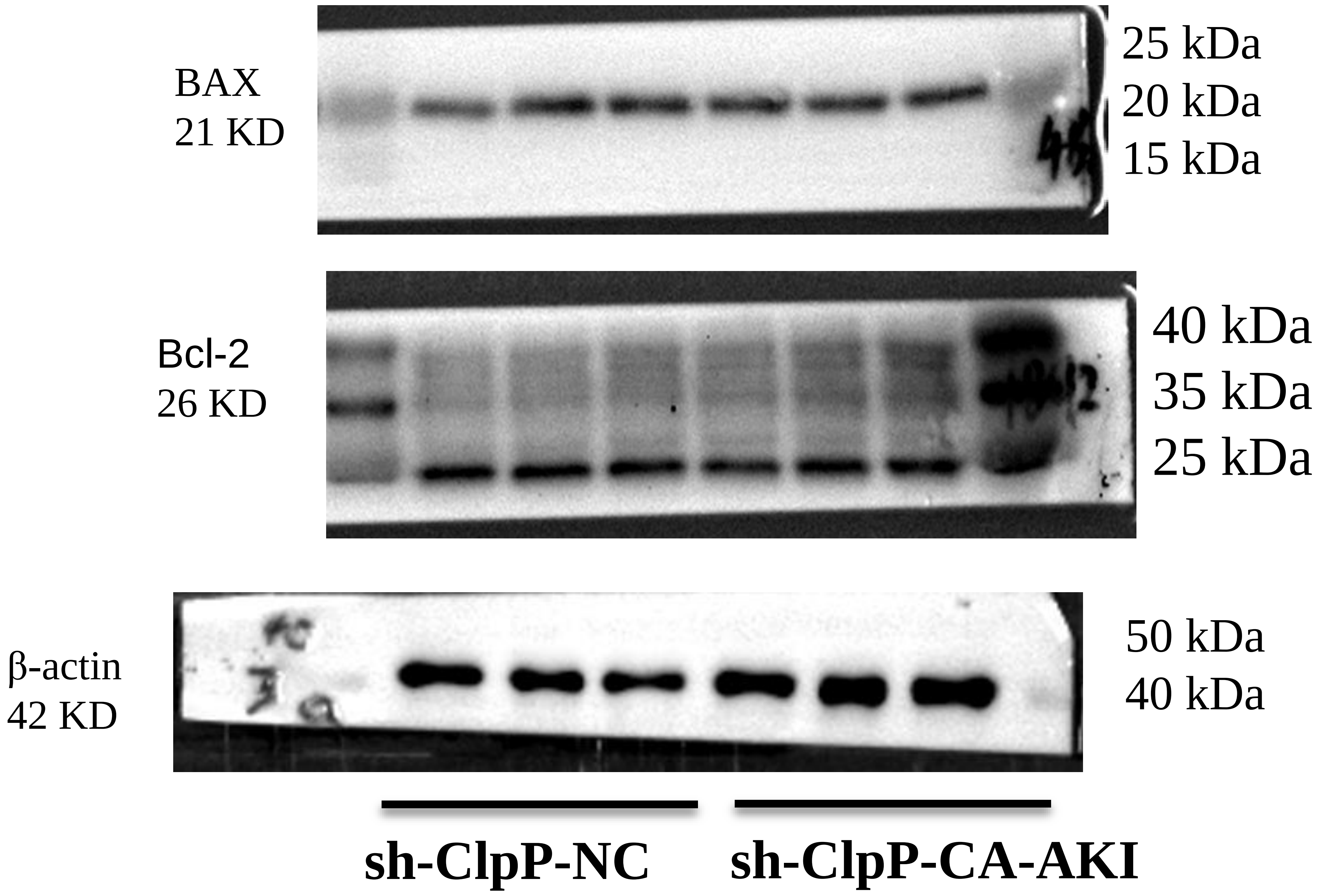
BAX
21 KD
25 kDa
20 kDa
15 kDa
Bcl-2
26 KD
40 kDa
35 kDa
25 kDa
β-actin
42 KD
50 kDa
40 kDa
sh-ClpP-NC
sh-ClpP-CA-AKI
