# Supplementary figures and images for: ClpP deficiency attenuates contrast-induced HK-2 cell injury through changes associated with mitochondrial dynamics and apoptosis
Source: PLoS One. 2026 Jul 2;21(7):e0352422. doi: 10.1371/journal.pone.0352422 (PMC13327117; doi:10.1371/journal.pone.0352422)

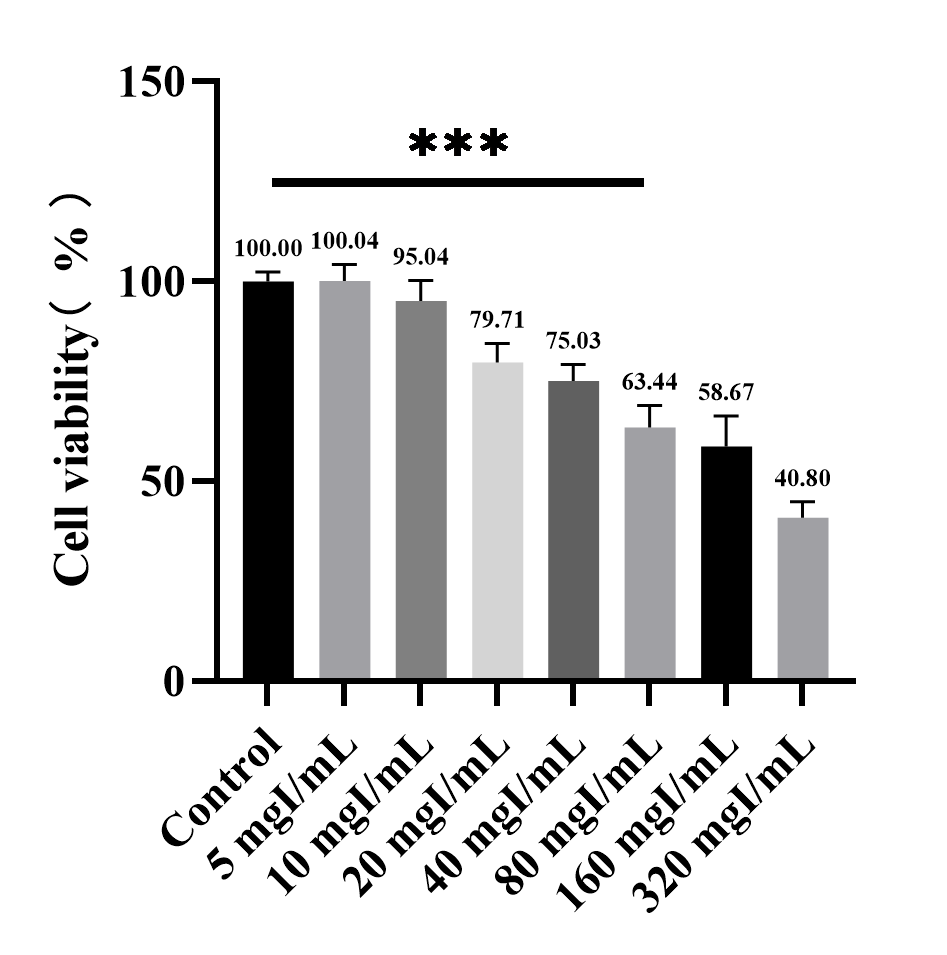

Supplement: S1 Fig — Cell viability was assessed by the CCK-8 assay. Iohexol at different concentrations (mg I/ml) caused decreased cell viability in a concentration-dependent manner. n = 6 in each group. Data are expressed as mean ± SD. *** P < 0.001. (TIF) [file pone.0352422.s002.tif]

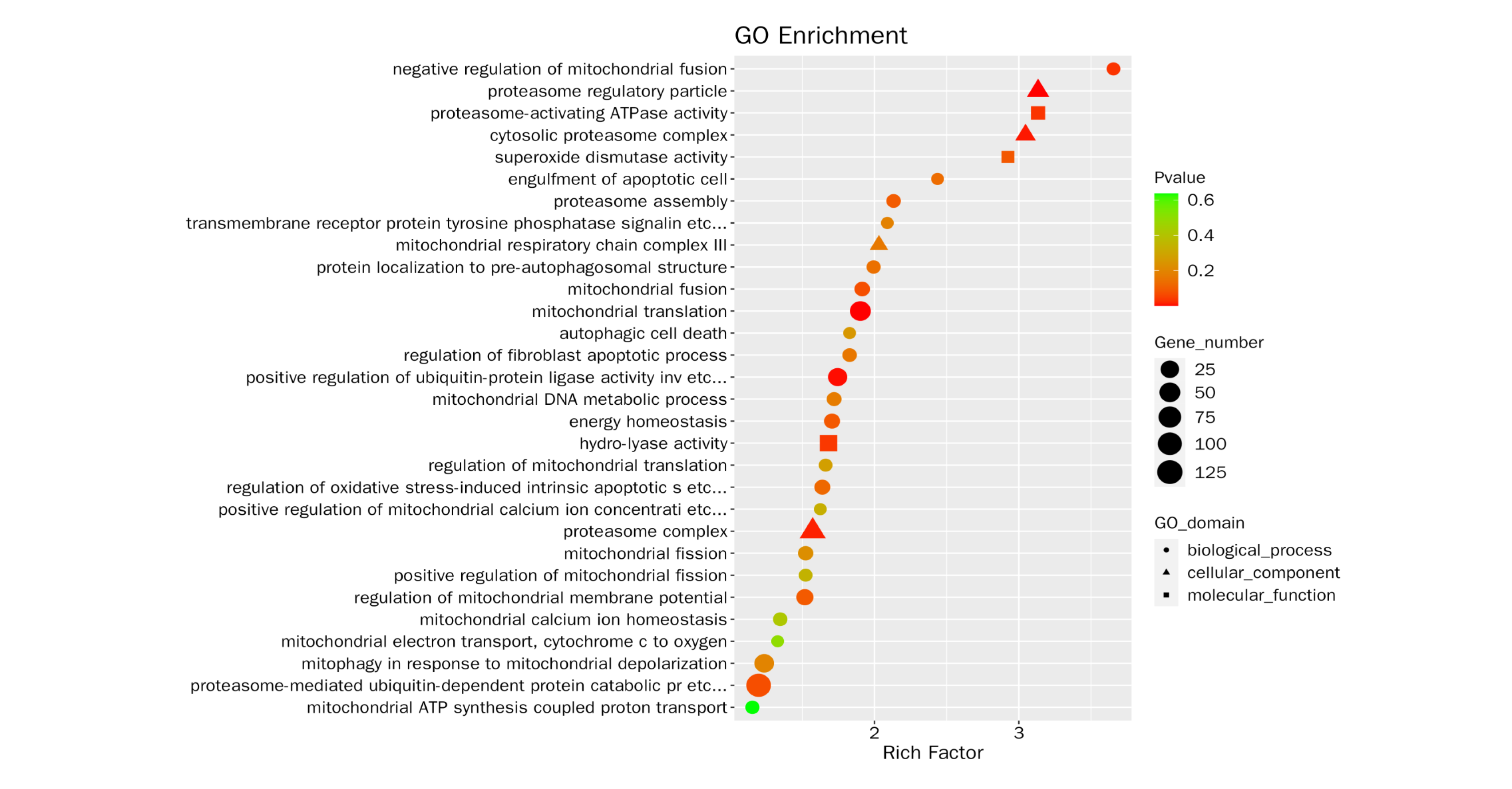

Supplement: S2 Fig — (TIF) [file pone.0352422.s003.tif]

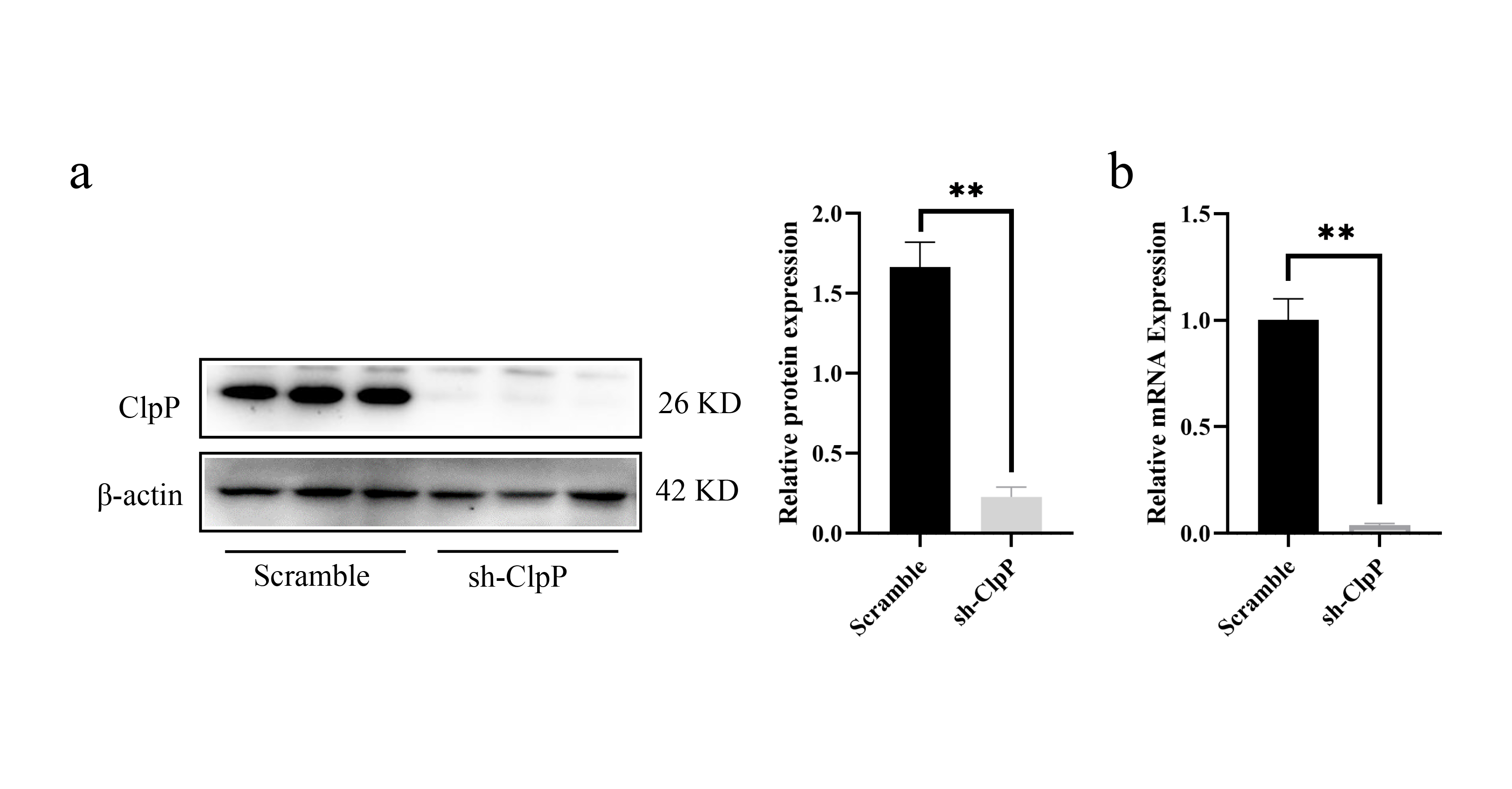

Supplement: S3 Fig — The mRNA and protein expression of ClpP in lentiviral-treated HK-2 cells are shown. (a) Representative Western blot analysis of ClpP in HK-2 cells, normalized to β-actin. (b) Relative expression of ClpP mRNA levels in HK-2 cells. n = 3 in each group. All data are shown as means ± SEM. ** P < 0.01. (TIF) [file pone.0352422.s004.tif]

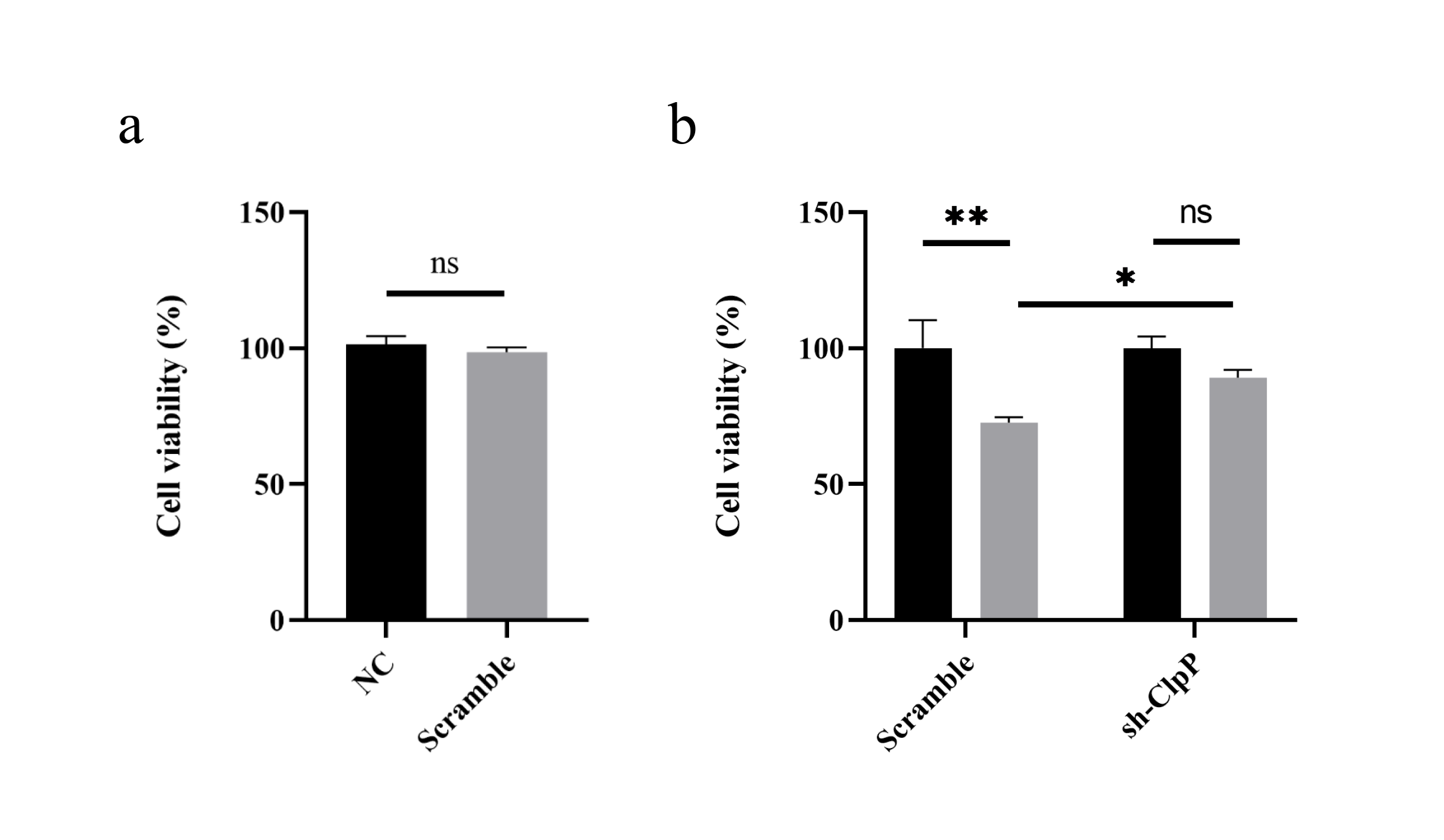

Supplement: S4 Fig — * P < 0.05, ** P < 0.01, *** P < 0.01.n = 3 in each group. NC, normal control. Data are shown as means ± SD. N.S. no significance. (TIF) [file pone.0352422.s005.tif]

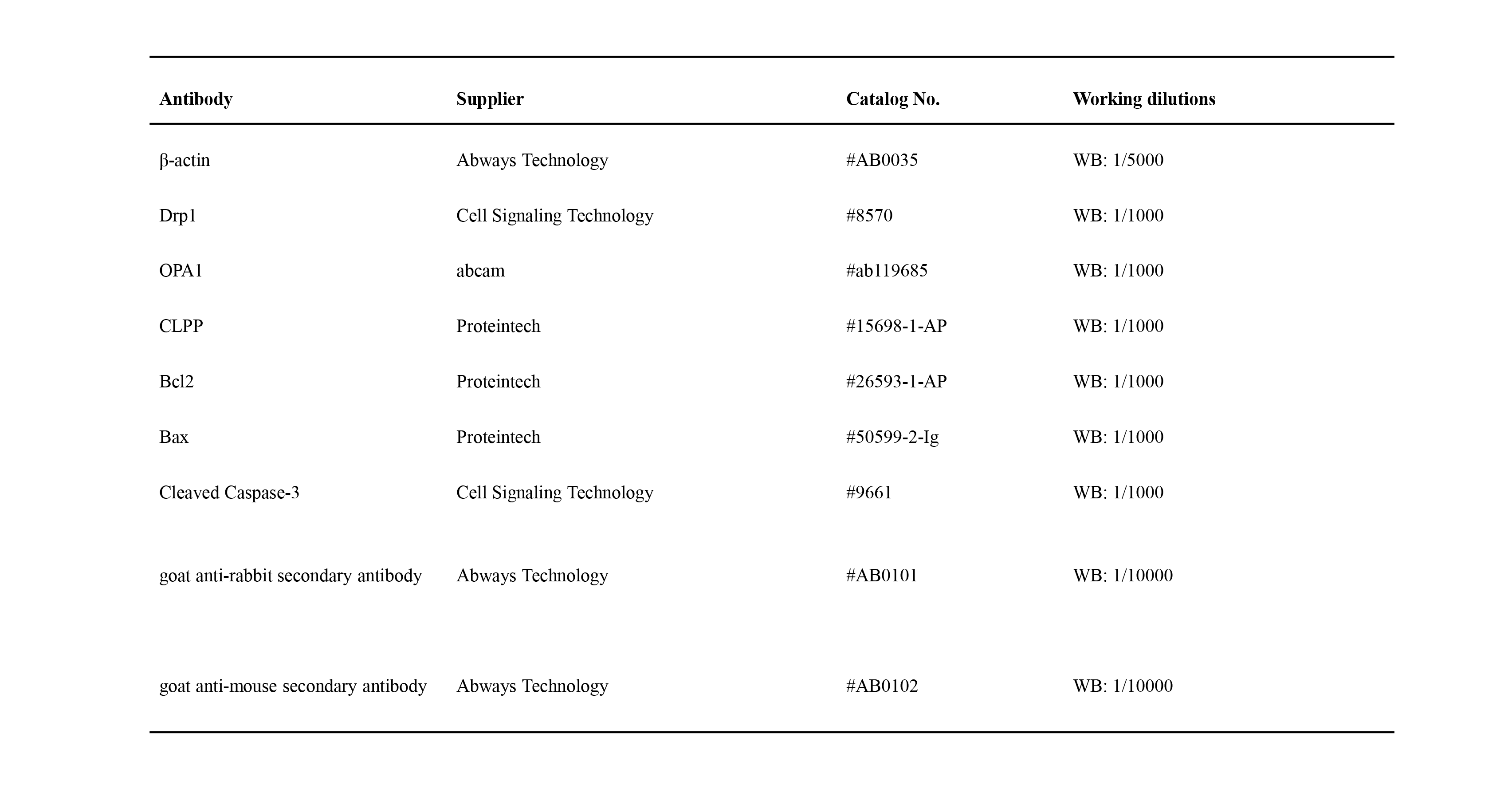

Supplement: S1 Table — (TIF) [file pone.0352422.s006.tif]
